# Contribution of Complex I NADH Dehydrogenase to Respiratory Energy Coupling in Glucose-Grown Cultures of *Ogataea parapolymorpha*

**DOI:** 10.1128/AEM.00678-20

**Published:** 2020-07-20

**Authors:** Hannes Juergens, Xavier D. V. Hakkaart, Jildau E. Bras, André Vente, Liang Wu, Kirsten R. Benjamin, Jack T. Pronk, Pascale Daran-Lapujade, Robert Mans

**Affiliations:** a Department of Biotechnology, Delft University of Technology, Delft, The Netherlands; b DSM Biotechnology Center, Delft, The Netherlands; c Amyris, Inc., Emeryville, California, USA; University of Toronto

**Keywords:** bioenergetics, bioreactor, chemostat, *Hansenula polymorpha*, NADH, P/O ratio, proteomics, respiration, retentostat, transcriptomics

## Abstract

The thermotolerant yeast Ogataea parapolymorpha (formerly Hansenula polymorpha) is an industrially relevant production host that exhibits a fully respiratory sugar metabolism in aerobic batch cultures. NADH-derived electrons can enter its mitochondrial respiratory chain either via a proton-translocating complex I NADH-dehydrogenase or via three putative alternative NADH dehydrogenases. This respiratory entry point affects the amount of ATP produced per NADH/O_2_ consumed and therefore impacts the maximum yield of biomass and/or cellular products from a given amount of substrate. To investigate the physiological importance of complex I, a wild-type O. parapolymorpha strain and a congenic complex I-deficient mutant were grown on glucose in aerobic batch, chemostat, and retentostat cultures in bioreactors. In batch cultures, the two strains exhibited a fully respiratory metabolism and showed the same growth rates and biomass yields, indicating that, under these conditions, the contribution of NADH oxidation via complex I was negligible. Both strains also exhibited a respiratory metabolism in glucose-limited chemostat cultures, but the complex I-deficient mutant showed considerably reduced biomass yields on substrate and oxygen, consistent with a lower efficiency of respiratory energy coupling. In glucose-limited retentostat cultures at specific growth rates down to ∼0.001 h^−1^, both O. parapolymorpha strains showed high viability. Maintenance energy requirements at these extremely low growth rates were approximately 3-fold lower than estimated from faster-growing chemostat cultures, indicating a stringent-response-like behavior. Quantitative transcriptome and proteome analyses indicated condition-dependent expression patterns of complex I subunits and of alternative NADH dehydrogenases that were consistent with physiological observations.

**IMPORTANCE** Since popular microbial cell factories have typically not been selected for efficient respiratory energy coupling, their ATP yields from sugar catabolism are often suboptimal. In aerobic industrial processes, suboptimal energy coupling results in reduced product yields on sugar, increased process costs for oxygen transfer, and volumetric productivity limitations due to limitations in gas transfer and cooling. This study provides insights into the contribution of mechanisms of respiratory energy coupling in the yeast cell factory Ogataea parapolymorpha under different growth conditions and provides a basis for rational improvement of energy coupling in yeast cell factories. Analysis of energy metabolism of O. parapolymorpha at extremely low specific growth rates indicated that this yeast reduces its energy requirements for cellular maintenance under extreme energy limitation. Exploration of the mechanisms for this increased energetic efficiency may contribute to an optimization of the performance of industrial processes with slow-growing eukaryotic cell factories.

## Abstract (precis)

Since popular microbial cell factories have typically not been selected for efficient respiratory energy coupling, their ATP yields from sugar catabolism are often suboptimal. In aerobic industrial processes, suboptimal energy coupling results in reduced product yields on sugar, increased process costs for oxygen transfer, and volumetric productivity limitations due to limitations in gas transfer and cooling. This study provides insights into the contribution of mechanisms of respiratory energy coupling in the yeast cell factory Ogataea parapolymorpha under different growth conditions and provides a basis for rational improvement of energy coupling in yeast cell factories. Analysis of energy metabolism of O. parapolymorpha at extremely low specific growth rates indicated that this yeast reduces its energy requirements for cellular maintenance under extreme energy limitation. Exploration of the mechanisms for this increased energetic efficiency may contribute to an optimization of the performance of industrial processes with slow-growing eukaryotic cell factories.

## INTRODUCTION

Crabtree-negative yeast species, which exhibit a respiratory sugar catabolism in aerobic batch cultures, are popular platforms for industrial production of proteins (1–5). The methylotrophic, Crabtree-negative yeasts Ogataea polymorpha and Ogataea parapolymorpha (6), both formerly known as Hansenula polymorpha, are popular protein expression platforms because of the availability of very strong but tightly controllable, methanol-inducible promoters. They are able to consume a wide range of carbon sources and assimilate nitrate, are thermotolerant up to 50°C, and exhibit fast, virtually by-product-free aerobic growth on glucose (7–9).

**FIG 1:**
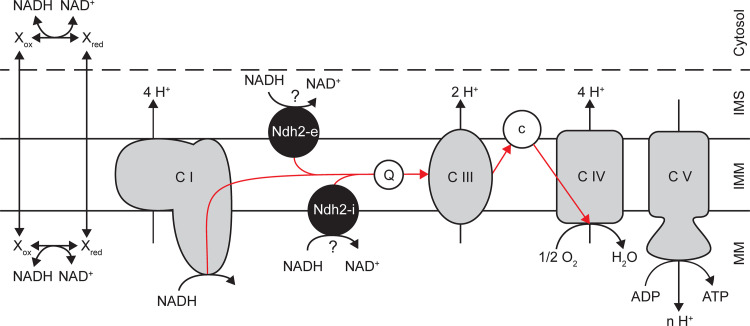
Putative respiratory chain structure of Ogataea (para)polymorpha illustrating routes to couple direct NADH oxidation to ATP formation. Respiratory complex I (C I) and possibly an internal alternative NADH dehydrogenase(s) (Ndh2-i) oxidize NADH in the mitochondrial matrix (MM). NADH generated in the cytosol can be directly oxidized by an external alternative NADH dehydrogenase(s) (Ndh2-e). Shuttles, consisting of a corresponding pair of cytosolic and mitochondrial dehydrogenases, might exist which can indirectly translocate NADH over the inner mitochondrial membrane (IMM). All NADH-oxidizing respiratory enzymes donate electrons (red arrows) to the quinone pool (Q), from which they are funneled linearly through the rest of the respiratory chain, consisting of complex III (C III), cytochrome *c* (C), and complex IV (C IV), before reduction of oxygen to water. Contrary to many other complex I-harboring yeasts, O. (para)polymorpha does not possess an alternative oxidase (15). Respiratory complexes I, III, and IV, but not Ndh2, contribute to the proton gradient across the inner mitochondrial membrane which is utilized by mitochondrial F_o_F_1_ ATPase, complex V (C V), for formation of ATP. The dashed line represents the outer mitochondrial membrane. IMS, intermembrane space.

Since formation of proteins and other nondissimilatory products from sugars requires a net input of cellular energy, efficient energy coupling of respiratory sugar catabolism is an important property of microbial protein production hosts. Respiratory energy coupling can be quantified by the P/O ratio, which represents the number of moles of ATP generated per mole of oxygen atoms reduced by the respiratory chain (10). In yeasts, the P/O ratio is dictated by the *in vivo* stoichiometries of electron transfer and proton translocation by respiratory chain complexes in the inner mitochondrial membrane, as well as by the stoichiometry of proton influx and ATP generation by the mitochondrial ATP synthase (11). Different respiratory chain components (Fig. 1) can overlap in their catalytic activities while exhibiting different stoichiometries of electron transfer and proton translocation, resulting in different P/O ratios (12). Whereas the canonical machinery for transfer of electrons from ubiquinone to oxygen (cytochrome *bc*_1_ complex or complex III and cytochrome *c* oxidase or complex IV) is strongly conserved among industrially relevant yeasts and fungi, major differences exist in coupling of the oxidation of mitochondrial NADH to the reduction of ubiquinone (13–15).

The respiratory chains of the industrially relevant yeasts Saccharomyces cerevisiae and Kluyveromyces lactis lack the large, multisubunit proton-translocating complex I NADH:ubiquinone oxidoreductase. Instead, they rely on a single-subunit, internal alternative NADH:ubiquinone oxidoreductase (Ndi1, generally referred to as internal alternative NADH dehydrogenase) that does not translocate protons (14, 16–18). Other yeasts and fungi, such as Yarrowia lipolytica (19), exclusively utilize complex I for respiratory oxidation of mitochondrial NADH or can express both complex I and an internal alternative NADH dehydrogenase, e.g., Neurospora crassa (20). O. polymorpha and its methylotrophic relative Pichia pastoris harbor complex I but also exhibit alternative NADH dehydrogenase activity. However, for the alternative NADH dehydrogenases, it is not known whether their catalytic sites for NADH oxidation face the mitochondrial matrix or the cytosol (21, 22) (Fig. 1).

In organisms which can synthesize both complex I and alternative NADH dehydrogenase(s), the relative contribution of these systems under industrially relevant conditions has not been fully elucidated. In several yeasts and fungi, studies performed with complex I inhibitors found that there was little or no impact on specific growth rates in batch cultures or that complex I activity was higher in late-exponential and/or stationary-phase cultures (21, 23–25), suggesting that the *in vivo* contribution of complex I may depend on the specific growth rate and/or substrate concentration. ATP from substrate catabolism is used to meet cellular maintenance energy requirements as well as formation of new biomass (26). Differences in respiratory energy coupling are therefore reflected in the biomass yield on the energy substrate (which serves as the electron donor for catabolism) (27). Since respiratory oxidation of NADH via complex I and alternative NADH dehydrogenase(s) results in different P/O ratios, the contribution of these systems can be assessed by quantitative analysis of biomass yields on sugar and oxygen of strains in which specific systems have been inactivated. Flux balance analysis simulations indicate that, in the absence of product formation and with a constant biomass composition, exclusive use of complex I for oxidation of mitochondrial NADH in respiratory cultures should result in a ca. 25% higher biomass yield on glucose than exclusive use of internal alternative NADH dehydrogenase (28).

Biomass yields and maintenance energy requirements of the yeasts S. cerevisiae and P. pastoris were previously studied under extreme glucose limitation in retentostat cultures (29–31). Retentostats are continuous cultivation setups with biomass retention in which biomass-specific rates of energy substrate consumption are progressively decreased until they eventually fulfill maintenance energy requirements of only metabolically active cells (32). Maintenance requirements include energy expenses for macromolecular turnover, membrane gradient homeostasis, and protective measures such as detoxification of reactive oxygen species (ROS) (33).

The aim of this study is to quantitatively assess the relative contribution of complex I and the alternative NADH dehydrogenases to respiratory oxidation of NADH, to biomass yields, and to maintenance energy metabolism in O. parapolymorpha. To this end, a complex I-deficient strain was constructed by disruption of the structural gene for the essential Nubm (51 kDa) subunit. The physiology of the mutant strain was then analyzed in aerobic, glucose-grown batch, chemostat, and retentostat cultures in bioreactors, covering a range of specific growth rates, and results were compared to data obtained with the congenic wild-type reference strain. In addition to quantitative analyses of biomass yields and maintenance energy requirements, strain- and condition-dependent adaptations were investigated by transcriptome and proteome analysis.

## RESULTS

### Disruption of complex I has a negligible effect on growth physiology in aerobic, glucose-grown batch cultures.

To assess under which cultivation conditions O. parapolymorpha utilizes complex I, a strain devoid of complex I activity was constructed. The structural gene encoding the essential, nuclearly encoded Nubm (51 kDa) subunit was disrupted in wild-type O. parapolymorpha strain CBS11895 by CRISPR/Cas9-assisted introduction of a single-nucleotide frameshift. Since Nubm, which is part of the peripheral N-module of complex I, contains the NADH-binding pocket (34), the frameshift mutation was expected to abolish complex I-mediated NADH oxidation and block entry of NADH-derived electrons into the enzyme. Whole-genome sequencing of the resulting strain, O. parapolymorpha IMD010, did not reveal any additional mutations in coding sequences.

For an initial physiological comparison, strain IMD010 and its congenic wild-type strain O. parapolymorpha CBS11895 were grown on glucose in aerobic bioreactor batch cultures. Under these glucose-excess conditions, both strains exhibited a specific growth rate of 0.37 h^−1^ (Table 1) and respiratory metabolism with negligible production of extracellular pyruvate (<0.1 mM), citrate (<0.1 mM), and ethanol (<1 mM). Biomass-specific glucose uptake rates and biomass yields of the two strains were not significantly different (Table 1). These observations indicated that complex I does not significantly contribute to NADH oxidation by O. parapolymorpha during aerobic batch cultivation on glucose.

**TABLE 1:** Physiology of wild-type strain O. parapolymorpha CBS11895 and complex I-disrupted mutant IMD010 in aerobic glucose-grown bioreactor batch cultures

Parameter^*a*^	Value for the parameter in:^*b*^		*P* value^*c*^
	CBS11895	IMD010	
μ (1/h)	0.37 ± 0.01	0.37 ± 0.00	0.78
*q*_Glucose_ (mmol glucose/g biomass)/h	−3.88 ± 0.02	−4.01 ± 0.00	0.11
*Y_X_*_/_*_S_* (g biomass/g glucose)	0.53 ± 0.01	0.52 ± 0.00	0.21

a μ, specific growth rate based on measurements of biomass dry weight concentration; *q*_Glucose_, biomass-specific glucose uptake rate during exponential growth phase; *Y_X_*_/_*_S_*, yield of biomass dry weight on glucose during exponential growth phase.

b Reported values are means ± standard errors of the means calculated from two independent cultures where errors smaller than the number of reported digits are rounded to 0.

c Reported *P* values (Student's *t* test) refer to the difference between mean values observed for CBS11895 and those for IMD010.

### Inactivation of complex I decreases biomass yields on glucose and oxygen in glucose-limited chemostat cultures.

To investigate the contribution of complex I at lower specific growth rates and at growth-limiting concentrations of glucose, the wild-type O. parapolymorpha strain CBS11895 and the complex I-deficient strain IMD010 were grown in aerobic, glucose-limited chemostat cultures (Table 2). At a dilution rate of 0.1 h^−1^, steady-state cultures of both strains exhibited a fully respiratory metabolism, without significant by-product formation and with residual glucose concentrations below 10 μM. Biomass protein contents of strains CBS11895 and IMD010 were 0.40 and 0.42 g of protein/g of biomass, respectively. Since the protein fraction of biomass accounts for the majority of the energetic costs of biosynthesis (35), this result indicates that the two strains exhibit similar energetic requirements for biomass formation (*Y_X_*_/ATP_). In agreement with earlier observations (9), the wild-type strain showed a biomass yield of 0.51 g of biomass/g of glucose. A 16% lower biomass yield on glucose for strain IMD010 indicated that, in the absence of a functional complex I, a larger fraction of the substrate needed to be respired to generate the same amount of ATP. Strain IMD010 also exhibited a 30% lower biomass yield on oxygen (Table 2), which, under the assumption of similar *Y_X_*_/ATP_ values and negligible maintenance energy requirements in these cultures, is equivalent to a 30% lower *in vivo* P/O ratio (23). Considering that alternative NADH dehydrogenases do not translocate protons and therefore are expected to conserve 40% less ATP than complex I (10, 13), the 30% lower *in vivo* P/O ratio is consistent with the phenotype expected when oxidation of mitochondrial NADH from glucose catabolism occurs via complex I in strain CBS11895 and is replaced by oxidation via alternative NADH dehydrogenase(s) in strain IMD010. Similar differences between strains CBS11895 and IMD010 were observed at a dilution rate of 0.025 h^−1^ (see Table S1 in the supplemental material).

**TABLE 2:** Physiology of wild-type O. parapolymorpha strain CBS11895 and complex I-disrupted mutant IMD010 in aerobic glucose-limited chemostat cultures at a dilution rate of 0.1 h^−1^

Parameter^*a*^	Value for the parameter in:^*b*^		*P* value^*c*^
	CBS11895	IMD010	
Actual dilution rate (1/h)	0.099 ± 0.000	0.100 ± 0.001	
Reservoir glucose (g/liter)	7.37 ± 0.02	7.40 ± 0.00	
Residual glucose (mM)	BDL	BDL	
*Y_X_*_/_*_S_* (g biomass/g glucose)	0.51 ± 0.00	0.43 ± 0.00	0.003
*Y*_X/O2_ (g biomass/g O_2_)	1.26 ± 0.01	0.88 ± 0.01	0.002
RQ	1.03 ± 0.00	1.03 ± 0.01	0.91
*q*_Glucose_ (mmol/g biomass)/h	−1.08 ± 0.01	−1.27 ± 0.01	0.005
*q*_CO2_ (mmol/g biomass)/h	2.52 ± 0.02	3.64 ± 0.04	0.005
*q*_O2_ (mmol/g biomass)/h	−2.44 ± 0.03	−3.54 ± 0.06	0.013
*C_X_* (g biomass/liter)	3.73 ± 0.02	3.21 ± 0.01	0.009
Protein content (g protein/g biomass)	0.40 ± 0.01	0.42 ± 0.00	0.22
Cell viability (%)	99.6 ± 0.0	99.9 ± 0.0	0.011
Carbon recovery (%)	99.5 ± 0.2	99.9 ± 0.4	0.53

a *Y_X_*_/_*_S_* and *Y_X_*_/O2_, yield of biomass dry weight on glucose and oxygen, respectively; RQ, respiratory quotient; *q*_Glucose_, *q*_CO2_, and *q*_O2_, biomass-specific uptake/production rates of glucose, CO_2_, and O_2_, respectively; *C_X_*, biomass dry weight concentration.

b Reported values are means ± standard errors of the means calculated from two independent steady-state cultures where errors smaller than the number of reported digits are rounded to 0. BDL, below the detection limit (10 μM). Reported cell viability is based on propidium-iodide staining. Carbon recovery calculations are based on a biomass carbon content of 48% (wt/wt).

c Reported *P* values (Student's *t* test) refer to the difference between mean values observed for CBS11895 and those for IMD010.

### O. parapolymorpha decreases its maintenance energy requirements at near-zero growth rates in retentostat cultures, independent of complex I.

Based on retentostat experiments with S. cerevisiae, maintenance energy requirements of yeasts were initially assumed to be growth rate independent (30, 31). This conclusion was in marked contrast with observations on several bacteria, in which a stringent response leads to decreased maintenance energy requirements at very low specific growth rates (36–38). Recent experiments on aerobic, glucose-limited retentostats of the Crabtree-negative yeast P. pastoris showed that, similarly, maintenance energy requirements at near-zero growth rates were approximately 3-fold lower than predicted from data obtained at higher specific growth rates (29). While these results indicate a stringent-response-like adaptation of non-*Saccharomyces* yeasts at near-zero growth rates, it is unclear whether this is related to their expression of a functional complex I NADH dehydrogenase. O. parapolymorpha, like P. pastoris, harbors both complex I and alternative NADH dehydrogenases, and we tested if a similar modulation of maintenance energy requirements occurred in O. parapolymorpha and if this was partly caused by a redistribution of respiratory flux to complex I.

Maintenance energy requirement (*m_S_*) and maximum theoretical biomass yield (*Y_X_*_/_*_S_*^max^) (26) values of O. parapolymorpha strains CBS11895 and IMD010 were first estimated from biomass-specific glucose uptake rates of aerobic, glucose-limited chemostat cultures grown at 0.025 h^−1^ and 0.1 h^−1^. Consistent with a lower energetic efficiency of the complex I-deficient strain IMD010, its estimated *m_S_* was higher (0.0241 ± 0.0008 versus 0.0142 ± 0.0008 [g glucose/g biomass]/h) and its *Y_X_*_/_*_S_*^max^ was lower (0.485 ± 0.003 versus 0.545 ± 0.003 g of biomass/g of glucose) than values obtained with the wild-type strain CBS11895 (Fig. S1).

**FIG 2:**
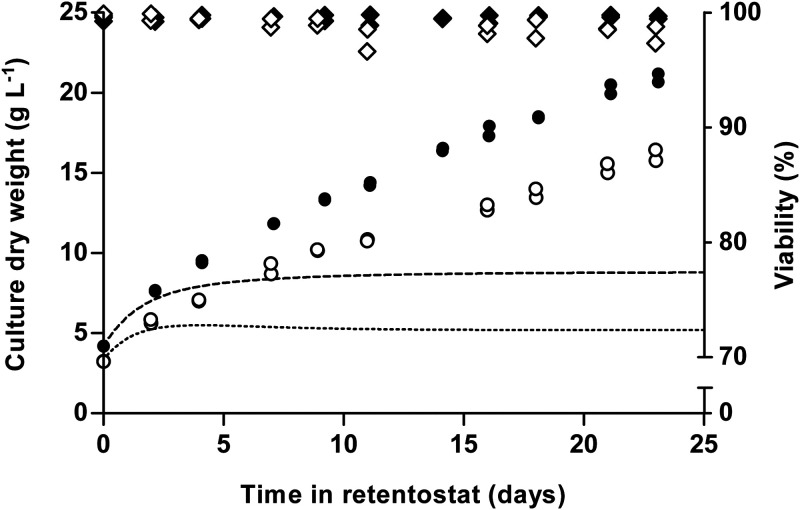
Biomass accumulation profile and viability of aerobic, glucose-limited retentostat cultures of wild-type O. parapolymorpha CBS11895 and the congenic complex I-deficient strain IMD010. The retentostat phase was initiated from steady-state chemostat cultures (*D* = 0.025 h^−1^) at time zero. Depicted are the measured biomass dry weight concentrations (circles) and culture viability based on propidium iodide staining (diamonds) of two independent cultures each of strains CBS11895 (closed symbols) and IMD010 (open symbols), as well as the predicted biomass accumulation profiles of CBS11895 (dashed line) and IMD010 (dotted line) based on *m_S_* and *Y_X_*_/_*_S_*^max^ values estimated from chemostat cultures grown at 0.1 and 0.025 h^−1^. The mean biomass concentration of CBS11895 was significantly higher than of that of IMD010 at each equivalent sampling point (Student's *t* test, *P < *0.05).

**FIG 3:**
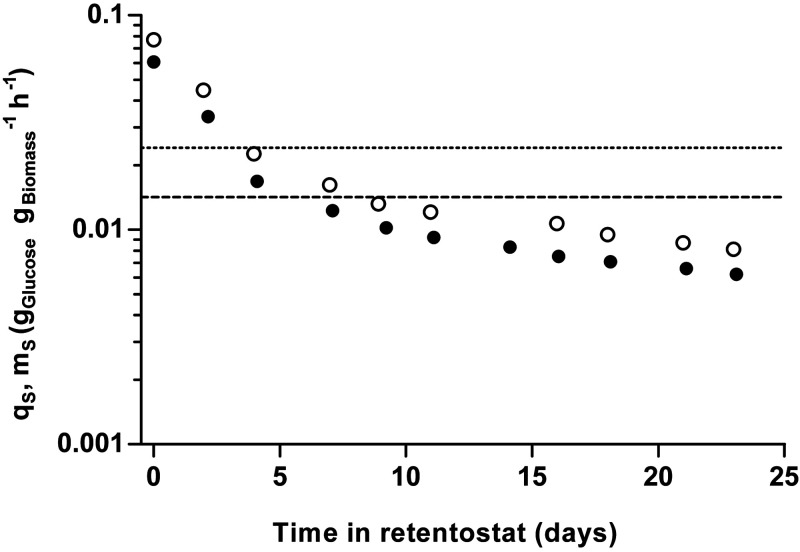
Biomass-specific glucose uptake rates (*q_S_*) during aerobic glucose-limited retentostat cultivation of wild-type O. parapolymorpha CBS11895 and the congenic complex I-deficient strain IMD010. Depicted *q_S_* values are means ± standard errors of the means (error bars smaller than symbol size) of two independent retentostat cultures each of CBS11895 (closed circles) and IMD010 (open circles) and were directly calculated from biomass accumulation. The values plotted at time zero correspond to the *q_S_* value in the steady-state chemostat cultures at 0.025 h^−1^ that preceded the retentostat cultures. Horizontal lines indicate the maintenance energy requirements (*m_S_*) calculated from chemostat cultures grown at 0.1 and 0.025 h^−1^ for strains CBS11895 (dashed) and IMD010 (dotted). With the exception of the values calculated at 7 and 9 days of cultivation, the *q_S_* values of CBS11895 were significantly lower than those of IMD010 at each equivalent sampling point (Student's *t* test, *P < *0.05).

**FIG 4:**
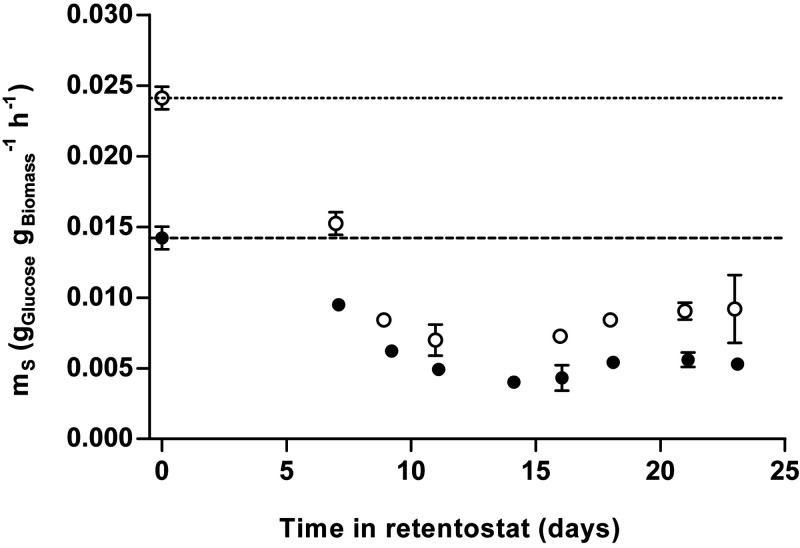
Depicted *m_S_* values are means ± standard errors of the means of two independent retentostat cultures each of strains CBS11895 (closed circles) and IMD010 (open circles) and were calculated via linear regression from sets of corresponding μ and *q_S_* values (directly calculated from biomass accumulation) from five adjacent sample points. The values plotted at time zero correspond to *m_S_* values determined by chemostat cultivation at 0.1 and 0.025 h^−1^, also represented by dashed (CBS11895) and dotted (IMD010) lines. With the exception of time points at 16 (CBS11895) as well as at 7 and 23 (IMD010) days of cultivation, *m_S_* values of both strains were found significantly lower than those determined by chemostat cultivation (Student's *t* test, *P < *0.05).

During 23 days of retentostat cultivation, specific growth rates of strains CBS11895 and IMD010 decreased to approximately 0.001 h^−1^, corresponding to a doubling time of over 28 days. As biomass concentrations in the retentostats increased and the specific growth rate progressively decreased, culture viability, measured by propidium iodide (PI) staining and CFU counts, remained near 100% (Fig. 2 and Fig. S2). At near-zero growth rates, the biomass protein content of CBS11895 decreased to 0.33 g of protein/g of biomass, while biomass composition of strain IMD010 remained the same as that observed in chemostat cultures at higher specific growth rates (Fig. S3). In the retentostats, the biomass-specific glucose uptake rates (*q_S_*) of both strains decreased below the *m_S_* values estimated from chemostat data, reaching 0.0062 ± 0.0001 and 0.0081 ± 0.0002 (g glucose/g biomass)/h at the end of retentostat cultivation for strains CBS11895 and IMD010, respectively (Fig. 3). By performing linear regression on sections of adjacent sample points, *m_S_* values were estimated throughout the retentostat cultivation runs from μ and *q_S_* values (Fig. 4). From this analysis, *m_S_* values of both strains were found to decrease during retentostat cultivation to values that were 2.5- to 3-fold lower than those estimated from chemostat data (Fig. 4). In accordance with an *m_S_* value that was lower than expected, biomass concentrations of CBS11895 during the retentostat cultivation increased above the maximum level predicted based on a growth rate-independent *m_S_* (Fig. 2). These observations demonstrated that at low specific growth rates, similar to P. pastoris, O. parapolymorpha exhibits a growth rate-dependent substrate requirement for maintenance. Since the complex I-deficient O. parapolymorpha strain IMD010 exhibited a decrease in maintenance energy requirements similar to that of the wild-type strain CBS11895 (Fig. 2), this adaptation is independent of the contribution of complex I to respiratory energy coupling.

### Transcriptional adaptations of O. parapolymorpha to lack of functional complex I.

To investigate whether the absence of the complex I Nubm subunit results in transcriptional adaptations in O. parapolymorpha, transcriptome sequencing (RNA-seq) was performed on samples taken from the glucose-grown bioreactor batch cultures, the chemostat cultures grown at dilution rates of 0.1 and 0.025 h^−1^, and from the late-stage retentostat cultures (samples taken after 23 days, at a specific growth rate of ca. 0.001 h^−1^) of strains CBS11895 and IMD010.

**FIG 5:**
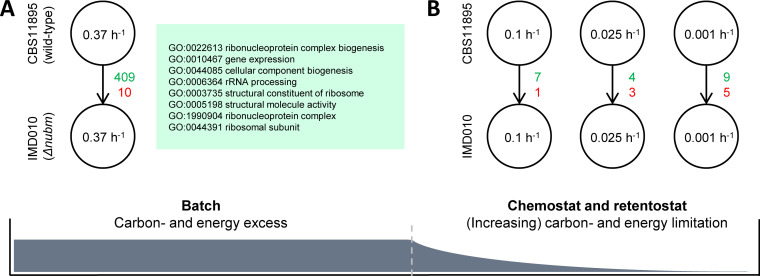
Transcriptional response of O. parapolymorpha to a lack of functional respiratory complex I. Green (upregulated) and red (downregulated) numbers indicate how many genes were found significantly differentially expressed (absolute log_2_ fold change of >2; FDR < 0.001) in strain IMD010 (disrupted complex I Nubm subunit) compared to levels in strain CBS11895 (wild type) in glucose-grown batch cultures (A) and glucose-limited chemostat (0.1 and 0.025 h^−1^) and late-stage retentostat cultures (0.001 h^−1^) (B). Boxed in green are the most highly enriched GO terms within the set of upregulated genes in IMD010 under batch conditions (based on 275 out of 409 genes for which an S. cerevisiae ortholog could be identified) (see Table S2 in the supplemental material for an extended list). Numbers inside circles represent the specific growth rate/dilution rate of the different cultures.

To focus on large changes in expression levels, genes were considered significantly differentially expressed between strains CBS11895 and IMD010 when the absolute log_2_ fold change of expression was larger than 2 and the false-discovery rate (FDR) was below 0.001. Whereas large differences in growth phenotypes were observed in glucose-limited chemostat and retentostat cultures, the absence of functional complex I only marginally affected the transcriptome of IMD010 grown under these conditions (Fig. 5B). However, while CBS11895 and IMD010 exhibited the same growth phenotypes in glucose-grown batch cultures, a large number of genes were significantly differentially expressed between the strains under these conditions, and most of these showed higher transcript levels in the complex I-deficient strain IMD010 (Fig. 5A). These 419 differentially expressed genes did not contain any of the known subunits of respiratory complex I. Among the set of 275 of the 410 upregulated genes for which an S. cerevisiae orthologue could be identified, the Gene Ontology (GO) terms related to organization and biogenesis of cellular components were enriched (Fig. 5A; see Table S2 for an extended list of enriched GO terms).

**FIG 6:**
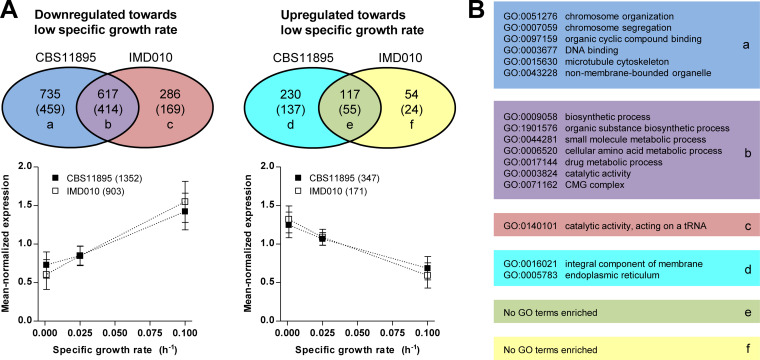
Transcriptional adaptation of wild-type O. parapolymorpha CBS11895 and congenic complex I-deficient strain IMD010 to increasingly lower specific growth rates. (A) The bottom graphs show mean-normalized expression levels of genes identified to be positively (left) and negatively (right) correlated with specific growth rates in strains CBS11895 and IMD010, based on samples taken from glucose-limited chemostat (0.1 and 0.025 h^−1^) and late-stage retentostat (0.001 h^−1^) cultures (data presented as means ± standard deviation). Venn diagrams at the top indicate the overlap between genes identified to be positively (left) and negatively (right) correlated with specific growth rates identified for CBS11895 and IMD010. Numbers in parentheses indicate genes for which an S. cerevisiae ortholog could be identified. (B) Significantly enriched GO terms identified in the sets of genes with growth rate-correlated expression. Colors and lowercase letters correspond to Venn diagrams in panel A. For each set, the two most highly enriched GO terms of a category (biological process, molecular function, and cellular component) are listed, except for set b, for which all significantly enriched GO terms are shown (see Table S3 in the supplemental material for extended list).

Absence of functional complex I also affected transcriptional adaptations to increasingly lower specific growth rates in glucose-limited cultivation regimes. A total of 1,699 and 1,074 genes whose transcript levels correlated positively or negatively with the specific growth rate in glucose-limited cultures were identified for strains CBS11895 and IMD010, respectively (Fig. 6A). For the majority of these genes, transcript levels correlated positively with the specific growth rate; i.e., expression was lower with low specific growth rates in both CBS11895 and IMD010 (Fig. 6A). Approximately two-thirds of the identified growth rate-correlated genes for strain IMD010 exhibited the same regulation in strain CBS11895, whereas the majority of the genes identified to be growth rate correlated in CBS11895 were specifically identified in this strain background (Fig. 6A). The response to the low specific growth rates of both CBS11895 and IMD010 was characterized by the transcriptional downregulation of genes involved in biosynthesis and metabolic processes as indicated by the enrichment of GO terms “biosynthetic process,” “cellular amino acid metabolic process,” and “catalytic activity” in this gene set (231, 54, and 263 out of 414 genes, respectively) (Fig. 6B; see Table S3 for an extended list of enriched GO terms). Among the genes that were positively correlated with the specific growth rate only in strain CBS11895, an enrichment of GO terms related to chromosome organization, DNA binding, and the cytoskeleton was observed, while the GO term “catalytic activity, acting on a tRNA” was enriched in the set of positively correlated genes unique to strain IMD010 (Fig. 6B; Table S3). Finally, among the smaller sets of genes which exhibited negative correlation of transcript levels with the specific growth rate in glucose-limited cultures, an enrichment of GO terms was detected only for the set of genes uniquely regulated in strain CBS11895 (related to integral membrane components and the endoplasmic reticulum) (Fig. 6B; Table S3).

### Condition-dependent redistribution of respiratory fluxes between complex I and alternative mechanisms for NADH (re)oxidation.

In glucose-limited chemostat cultures, the complex I-deficient strain O. parapolymorpha IMD010 exhibited a lower biomass yield on substrate and oxygen than the wild-type strain CBS11895 but retained a fully respiratory metabolism. These observations indicated that glucose-limited cultures of strain IMD010 employed an alternative, energetically less efficient mechanism(s), such as alternative NADH dehydrogenase(s), to replace the role of complex I in NADH oxidation. Based on sequence homology to known alternative NADH dehydrogenases and the C-terminal domain unique to this class of enzymes (39), the genome of O. parapolymorpha was predicted to encode three alternative NADH dehydrogenases (Fig. S4), here referred to as Ndh2-1, Ndh2-2, and Ndh2-3 (encoded by HPODL_02792, HPODL_00256, and HPODL_02018, respectively). Depending on substrate specificity and localization on the inner mitochondrial membrane, each of these enzymes could potentially contribute to reoxidation of NADH generated in the mitochondrial matrix.

To investigate condition-dependent expression of these alternative dehydrogenases, their protein abundance levels and those of complex I subunits were determined by mass spectrometry (MS)-based proteomics analysis on samples taken from the glucose-grown batch, chemostat (dilution rate of 0.1 and 0.025 h^−1^), and late-stage retentostat cultures (specific growth rate of ca. 0.001 h^−1^) for strains CBS11895 and IMD010. Proteomics analysis of these samples detected 1,351 O. parapolymorpha proteins with high combined detection confidence (FDR of <1%), including the three alternative NADH dehydrogenases as well as nearly all subunits of complex I (see Data Set S1 for protein abundance data).

**FIG 7:**
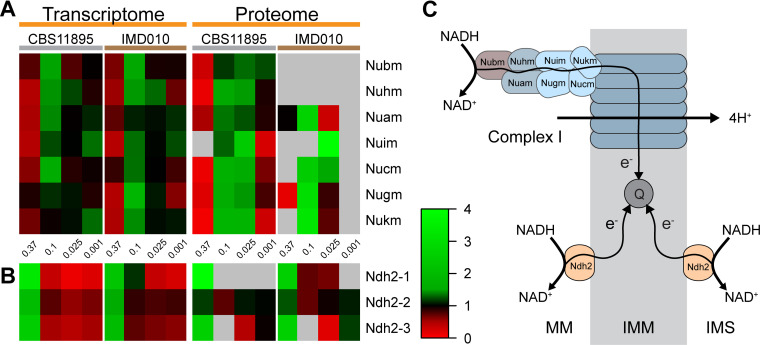
Mean-normalized transcript and protein abundances of essential complex I subunits (A) and alternative NADH dehydrogenases (B) in O. parapolymorpha strains CBS11895 (wild type) and IMD010 (disrupted complex I Nubm subunit). Samples were taken from duplicate independent aerobic, glucose-grown batch (0.37 h^−1^), chemostat (0.1 and 0.025 h^−1^), and late-stage retentostat (0.001 h^−1^) cultures. Transcript and protein abundances were mean normalized separately for each gene and strain. Gray, protein not detected based on criteria described in Materials and Methods. (C) The location and catalyzed reactions of the enzymes. Localization of the three alternative NADH dehydrogenases is unknown, and any of the enzymes could be internally (MM) or externally (IMS) localized. MM, mitochondrial matrix; IMM, inner mitochondrial membrane; IMS, intermembrane space.

Mean-normalized transcript and protein abundance levels of the three alternative NADH dehydrogenases were compared to those of the seven essential nuclearly encoded subunits of complex I to investigate their strain- and condition-dependent expression (Fig. 7). In strains CBS11895 and IMD010, transcript levels of the seven complex I subunits were on average 2.3- and 2.6-fold lower, respectively, in glucose-grown batch cultures than in chemostat cultures grown at a dilution rate of 0.1 h^−1^. Differences in protein levels of these subunits were more pronounced. In batch cultures of strain CBS11895, these subunits were less abundantly detected, and protein levels were on average 11.3-fold lower than in the chemostat cultures while most subunits were not detected at all in batch cultures of strain IMD010 (Fig. 7; Table S4). In addition to the disrupted Nubm subunit, the Nuhm (24 kDa) subunit of complex I was not detected in any of the proteome analyses on strain IMD010. In contrast to the changes in expression of complex I, in both O. parapolymorpha strains the three alternative NADH dehydrogenases Ndh2-1, Ndh2-2 and Ndh2-3 consistently showed higher transcript and protein levels in batch cultures than in chemostat cultures grown at 0.1 h^−1^ (Fig. 7). These observations are consistent with the similar growth characteristics of strains CBS11895 and IMD010 in glucose-grown batch cultures and support the conclusion that complex I plays an insignificant role in respiratory NADH reoxidation by O. parapolymorpha at high glucose concentrations.

Transcript levels of complex I subunits in retentostat cultures were similar to those in chemostat cultures, while the corresponding proteins were less abundantly detected and exhibited lower protein levels. In strain CBS11895, protein levels of the represented essential complex I subunits were on average 3.5-fold lower in late-stage retentostats than in chemostat cultures grown at a dilution rate of 0.025 h^−1^. In strain IMD010, most complex I subunits, including the numerous accessory subunits, were not detected under these conditions (Fig. 7; Table S4).

## DISCUSSION

### Contribution of complex I to respiratory glucose metabolism and growth energetics.

Physiological analysis of O. parapolymorpha CBS11895 and its congenic mutant IMD010 showed that the complex I NADH dehydrogenase does not play a major role in respiratory NADH oxidation in aerobic, glucose-grown batch cultures. The reported insensitivity to complex I inhibitors of respiratory rates of aerobic, glucose-grown batch cultures of Candida utilis and Dekkera bruxellensis (23, 25) suggests that the physiological role and regulation of complex I in these facultatively fermentative yeasts resemble that in O. parapolymorpha. In contrast, complex I is essential for growth of the respiratory yeast Yarrowia lipolytica (12) while in the filamentous fungi Neurospora crassa and Aspergillus niger, its absence negatively affects specific growth rates and/or biomass yields in aerobic batch cultures (34, 40).

While aerobic, glucose-limited chemostat cultures of the complex-I deficient strain IMD010 showed lower biomass yields on glucose and oxygen than the reference strain CBS11895, they still exhibited a fully respiratory metabolism. Clearly, another mechanism for oxidation of mitochondrial NADH, with a lower ATP yield from oxidative phosphorylation, compensated for the absence of a functional complex I in these cultures. Since NADH cannot permeate the inner mitochondrial membrane (41), O. parapolymorpha additionally requires a mechanism for respiratory oxidation of NADH generated in the cytosol by glycolysis. By analogy of the situation in S. cerevisiae and other fungi (42, 43), it therefore seemed likely that the O. parapolymorpha genome encodes external alternative NADH dehydrogenase in combination with an NADH shuttle mechanism and/or a matrix-oriented (internal) alternative NADH dehydrogenase. For example, an ethanol-acetaldehyde shuttle in S. cerevisiae has been shown to shuttle electrons from mitochondrial NADH to the cytosol (44). Such a shuttle requires cytosolic and mitochondrial isoenzymes of NAD-linked alcohol dehydrogenase, which are both present in O. parapolymorpha (45, 46).

Transcript levels of a gene encoding a putative alternative NADH dehydrogenase (Ndh2-1) were significantly higher in glucose-limited chemostat cultures (dilution rate of 0.1 h^−1^) of O. parapolymorpha IMD010 than in the wild type (log_2_ fold change of 2.2). Moreover, Ndh2-1 was detected in the proteome of strain IMD010 grown under these conditions but not in that of strain CBS11895. These observations are consistent with Ndh2-1 being an internal, non-proton-translocating NADH dehydrogenase that can compensate for the absence of functional complex I in the mutant strain IMD010 and in batch cultures of the wild-type strain CBS11895. Coexistence of internal alternative NADH dehydrogenase and complex I has been observed in other fungi, including N. crassa (20), C. utilis (23), and A. niger (47).

Proteome and transcriptome analysis of respiratory complex I subunits and alternative NADH dehydrogenases in O. parapolymorpha CBS11895 indicated a switch from energy-efficient respiration via complex I in glucose-limited chemostat cultures to less efficient respiration via alternative NADH dehydrogenases in fast-growing batch cultures. It has been previously suggested that respiratory NADH oxidation by a simple single-subunit NADH dehydrogenase instead of the large multisubunit complex I may be beneficial when energy substrate is abundantly available (12). Expressed per amount of protein, alternative NADH dehydrogenases are likely to allow for faster oxidation of NADH than the multisubunit complex I. A switch to the energetically less efficient alternative dehydrogenases is therefore consistent with a strategy in which metabolic rates are maximized under substrate excess while energy substrate limitation is coupled to optimization of energy efficiency (48, 49). Similar trade-offs involving pathways with a high ATP yield but high protein cost (i.e., low protein efficiency) have been implicated in overflow metabolism in Escherichia coli, S. cerevisiae, and human muscle cells (50, 51).

Analysis of late-stage retentostat cultures suggested a higher utilization of alternative NADH dehydrogenases at extremely low specific growth rates than in faster chemostat cultures. Condition-dependent use of the different NADH dehydrogenases has been suggested to reflect the need to balance energy demand with NAD^+^ regeneration (13, 52) and to prevent reactive oxygen species (ROS) formation, potentially by altering the degree of reduction of the quinone pool (43, 53). Alternative, less efficient respiratory pathways are widespread in yeast (52, 54, 55), and examples exist of species that redirect flux through these pathways under low-energy-substrate conditions to limit ROS formation (56, 57). Apparently, in these organisms decreased ROS formation can outweigh the benefits of increased energetic efficiency under these conditions, and a similar mechanism could be beneficial for long-term survival in O. parapolymorpha at very low specific growth rates as well. Indeed, investigation of the expression of catalase as well as candidate genes for superoxide dismutase and cytochrome *c* peroxidase demonstrated that out of the five genes for which protein abundances could reliably be detected, four were more abundantly expressed at low specific growth rates (see Fig. S5 in the supplemental material).

### Regulation of complex I expression.

Under several of the tested conditions, many essential complex I subunits were not detected in proteome analyses on the mutant strain IMD010. In this strain, the Nuhm (24 kDa) subunit was not even detected in chemostat samples in which other complex I subunits were most abundant. In normal complex I assembly, Nubm and Nuhm, which are both part of the complex I N-module, preassemble into a stable heterodimer which is retained even after breakdown of complex I after inhibition of mitochondrial translation (58). Destabilization of Nuhm caused by deletion of the *NUBM* gene would resemble observations on human complex I, which showed that loss of individual subunits affected protein abundance of other subunits from the same structural module (59).

In mammals, plants, and fungi such as Y. lipolytica and N. crassa, complex I forms respiratory supercomplexes with complexes III and IV (60–63). A study on mammalian cells did not detect formation of supercomplexes with a complex I lacking the Nubm-containing N-module (58). However, the fast respiratory growth of strain IMD010 indicated that, even in the complete absence of the Nubm subunit, O. parapolymorpha expressed a functional respiratory chain. If required for respiration (64), respiratory supercomplexes in strain IMD010 might resemble the supercomplex-like structures observed in S. cerevisiae, which are comprised of complexes III and IV with the internal alternative NADH dehydrogenase Ndi1 (65).

### Maintenance energy requirements in O. parapolymorpha.

Similar to observations in P. pastoris (29) and several bacteria (36–38), O. parapolymorpha modulated its substrate requirements for cellular maintenance (*m_S_*) in a growth rate-dependent manner. At near-zero growth rates, substrate consumption rates were substantially lower than estimated from faster-growing chemostat cultures. Independent of the specific growth rate and in line with the higher P/O ratio enabled by involvement of complex I in respiratory oxidation of NADH, the wild-type strain CBS11895 exhibited lower maintenance energy requirements than the complex I-disrupted strain IMD010 in all glucose-limited chemostat and retentostat cultures. However, complex I did not play a role in the modulation of *m_S_* at low specific growth rates.

Similar to previous work with P. pastoris (29), analysis of the transcriptome data revealed two regulatory patterns in O. parapolymorpha: gene expression that correlated negatively and gene expression that correlated positively with the specific growth rate. In contrast to P. pastoris, the majority of these genes were found to correlate positively with a specific growth rate in O. parapolymorpha and in both strain CBS11895 and IMD010 indicated reduced expression levels of biosynthesis-related genes toward lower growth rates. We were not able to relate these growth rate-correlated genes to the mechanisms responsible for the stringent-response-like response in O. parapolymorpha. However, because many genes of this yeast lack functional annotation as they are unique to O. (para)polymorpha or a small clade of neighboring yeasts, we limited our analysis to ca. 60% of genes that have orthologs in S. cerevisiae, a yeast that does not display a stringent-response-like adaptation at near-zero growth rates (30, 31). Therefore, mining the subset of genes for which no S. cerevisiae ortholog is known could provide novel insights into the mechanism behind the eukaryotic stringent response.

## MATERIALS AND METHODS

### Yeast strains, culture conditions, and maintenance.

The yeast strains used in this study were CBS11895 (DL-1), a wild-type Ogataea parapolymorpha strain ordered from CBS-KNAW (Westerdijk Fungal Biodiversity Institute, Utrecht, The Netherlands), and IMD010, a CBS11895-derived mutant with a disruption in the *NUBM* gene (*nubm^G445GC^*). Strains were grown in an Innova shaker incubator (New Brunswick Scientific, Edison, NJ, USA) set to 30°C and 200 rpm, in 500-ml shake flasks containing 100 ml of medium. Heat-sterilized (120°C for 20 min) YPD medium (10 g liter^−1^ Bacto yeast extract, 20 g liter^−1^ Bacto peptone, 20 g liter^−1^ glucose, demineralized water) was used for strain construction and maintenance. Solid medium was prepared by addition of 2% (wt/vol) agar to YPD medium. Frozen stock cultures were prepared from exponentially growing shake flask cultures by addition of glycerol to a final concentration of 30% (vol/vol) and aseptically stored in 1-ml aliquots at −80°C.

### Molecular biology techniques and strain construction.

Escherichia coli strain DH5α was used for plasmid transformation, amplification, and storage. Plasmids were isolated from E. coli using a GenElute Plasmid Miniprep kit (Sigma-Aldrich, St. Louis, MO, USA). Genomic DNA of yeast colonies used as the template for diagnostic PCR was isolated using the lithium acetate (LiAc)-sodium dodecyl sulfate method (66). Diagnostic PCR was performed using DreamTaq polymerase (Thermo Fisher Scientific, Waltham, MA, USA) and desalted primers (Sigma-Aldrich). DNA fragments obtained by PCR were separated by gel electrophoresis, and PCR purification was performed with a GenElute PCR Clean-Up kit (Sigma-Aldrich).

For the construction of strain IMD010 (*O. parapolymorpha nubm*^G445GC^), the *O. parapolymorpha* open reading frame (ORF) encoding the complex I *NUBM* 51-kDa subunit (*OpNUBM*, locus tag HPODL_04625; GenBank accession number XM_014080963.1) was identified. *OpNUBM* was found via a homology search (blastn [https://blast.ncbi.nlm.nih.gov]) in the O. parapolymorpha CBS11895 (DL-1) RefSeq assembly (NCBI accession number GCF_000187245.1) (67) using the O. polymorpha (Pichia angusta) partial sequence identified as coding for the complex I Nubm subunit (GenBank accession no. AL434382) (21, 68) as input. Other O. parapolymorpha complex I subunits were assigned based on protein sequence homology (tblastn [https://blast.ncbi.nlm.nih.gov]) with known P. pastoris subunits (67, 69, 70). *OpNUBM* was disrupted using the pUDP CRISPR/Cas9 system described previously (71). The guide RNA (gRNA) donor plasmid pUD676 was *de novo* synthesized by GeneArt (Thermo Fisher Scientific) and contained the synthetic 233-bp double-stranded DNA (dsDNA) gRNA construct with a gRNA spacer sequence (5′-CCTGATGTAAATATACGCTG-3′) targeting *OpNUBM* after bp 445 out of 1,467 bp. pUD676 was then integrated into pUDP002 via BsaI-mediated assembly as described previously (71), yielding *OpNUBM*-targeting plasmid pUDP084. For disruption of *OpNUBM*, wild-type O. parapolymorpha was transformed with pUDP084 via electroporation and subjected to a prolonged liquid incubation protocol, as described previously for deletion of *OpADE2* and *OpKU80* (71). Primers 12200 and 12201 (5′-CCCAGCTACGATCTCAAGAC-3′ and 5′-AACTTGGTGCCCGAGTTAC-3′, respectively) were then used for PCR amplification of the *OpNUBM* locus of seven randomly picked single colonies, and subsequent Sanger sequencing (Baseclear, Leiden, The Netherlands) revealed that three out of seven tested colonies contained an indel at the gRNA target site. A single colony of one of the mutants, containing a single cysteine nucleotide insertion between position 445 and 446 of the *OpNUBM* ORF, was restreaked three times subsequently on nonselective YPD medium to remove pUDP084 and renamed IMD010.

### Bioreactor cultivation.

Bioreactor cultivation was performed using synthetic medium (SM) with the addition of 0.15 g liter^−1^ of Pluronic 6100 PE antifoaming agent (BASF, Ludwigshafen, Germany). SM was prepared according to C. Verduyn et al. (72) and autoclaved at 120°C for 20 min. Glucose and vitamins (72) were prepared separately and filter sterilized (vitamins) or heat sterilized at 110°C for 20 min (glucose). Bioreactors were inoculated with exponentially growing cells from independent shake flask cultures (grown as described above) with SM and 20 g liter^−1^ of glucose. All cultures were performed in 2-liter benchtop bioreactors (Applikon, Delft, The Netherlands) with initial volumes of 1.4 liters (batch) and working volumes of 1.0 or 1.4 liters (chemostat) or 1.4 liters (retentostat). Cultures were sparged with dried, compressed air (0.5 vvm [volume of gas per volume of liquid per minute]) and stirred at 800 rpm. Temperature was maintained at 30°C, and pH was controlled at 5.0 by automatic addition of a 2 M KOH (batch and chemostat) or 10% (wt/vol) NH_4_OH (retentostat) solution by an ADI 1030 Bio Controller system (Applikon) or by an ez-Control bioreactor controller (Applikon). In chemostats and retentostats, the working volume was maintained by an electrical level sensor that controlled the effluent pump. Culture exhaust gas from bioreactors was cooled with a condenser (2°C) and dried with a Perma Pure dryer (Inacom Instruments, Veenendaal, The Netherlands) prior to online analysis of carbon dioxide and oxygen with a Rosemount NGA 2000 analyzer (Emerson, St. Louis, MO, USA). Batch cultures were performed with 7.5 g liter^−1^ of glucose as the sole carbon source and an initial optical density at 660 nm (OD_660_) of 0.3 (approximately 0.05 g liter^−1^ of biomass dry weight). In chemostat cultures, 7.5 g liter^−1^ of glucose was used as a sole carbon source, and the dilution rate (*D*) was set by maintaining a constant inflow rate. Cultures were assumed to have reached a steady state when, after a minimum of 5 volume changes, the oxygen consumption rate, carbon dioxide production rate, and biomass concentration changed by less than 3% over two consecutive volume changes. Retentostat cultivation was performed essentially as described by C. Rebnegger et al. (29). To predict the accumulation of biomass in retentostat cultures of strains CBS11985 and IMD010, the predictive biomass accumulation script of C. Rebnegger et al. (29) was used with *m_S_* and *Y_X_*_/_*_S_*^max^ values as estimated from chemostat cultures with *D* values of 0.1 and 0.025 h^−1^. Based on the assumption that maintenance energy requirements are growth rate independent, a feeding regime was selected for O. parapolymorpha in which 10 g liter^−1^ of glucose was used for the preceding chemostat phase, and 5 g liter^−1^ was used for the retentostat phase in combination with a 1.2-liter mixing vessel, which was identical to the setup used for the earlier work on retentostat cultivation of P. pastoris (29). The dilution rate was determined by maintaining a constant inflow rate of medium from the mixing vessel, a 3-liter benchtop bioreactor (Applikon) with a working volume of 1.2 liters, and stirred at 500 rpm. The volume in the mixing vessel was kept constant by an electrical level sensor that controlled the feed pump of the mixing vessel. The medium was SM as described above but contained an additional 0.5 ml liter^−1^ of the concentrated trace element solution (1.5× final concentration) and an additional 1 ml liter^−1^ of the vitamin stock solution (2× final concentration) (72). Retentostat cultivation was preceded by a chemostat cultivation (*D* of 0.025 h^−1^) using the same conditions as described for the subsequent retentostat cultivation. During the chemostat phase, the medium flowing into the mixing vessel contained 10 g liter^−1^ of glucose. Once a steady state was achieved, the retentostat phase was initiated by two changes: (i) the medium flowing into the mixing vessel was changed to be drawn from a medium vessel with identical medium composition but with 5 g liter^−1^ of glucose as the limiting compound, and (ii) the culture effluent was redirected through a filtered effluent port, equipped with a hollow stainless steel filter support with an autoclavable hydrophobic polypropylene filter with 0.22-μm pore size (Trace Analytics, Braunschweig, Germany). Prior to heat sterilization, the filter was soaked overnight in a 96% ethanol solution and subsequently rinsed with 1× phosphate-buffered saline (Sigma-Aldrich).

### Biomass measurements.

Optical density was measured at 660 nm on a Jenway 7200 spectrophotometer (Jenway, Staffordshire, UK). For biomass dry weight determination (typically performed in triplicate), exactly 10 ml of culture broth was filtered over predried and preweighed membrane filters (0.45-μm pore size; Pall Corporation, Ann Arbor, MI, USA), which were washed with demineralized water, dried in a microwave oven at 350 W for 20 min, and weighed immediately (73). Samples from chemostat and retentostat cultures were diluted with demineralized water prior to filtration to obtain a biomass dry weight concentration of approximately 2 g liter^−1^. The exact dilution was calculated by weighing the amount of sample and diluent and assuming a density of 1 g ml^−1^ for both fractions. For samples from late-stage retentostat cultures of strain IMD010 (after approximately 14 days and onwards), membrane filters were placed in glass bowls and covered with plastic funnels for microwave drying as under normal drying conditions biomass flakes formed that detached from the membrane filters, preventing accurate determination. Membrane filters were routinely redried and reweighed to ensure complete drying. Biomass protein content was determined using dried bovine serum albumin (BSA) (fatty acid free; Sigma-Aldrich) as described previously (74), with the modifications that NaOH was used instead of KOH and absorbance was measured at 510 instead of at 550 nm. Culture samples were diluted with demineralized water to biomass dry weight concentrations between 2.2 and 3.8 g liter^−1^ prior to protein content analysis.

### Metabolite analysis.

For the determination of extracellular metabolite concentrations during batch fermentations, 1-ml aliquots of culture sample were centrifuged for 3 min at 20,000 × *g*, and the supernatant was used for analysis. Samples from chemostat and retentostat cultures were rapidly quenched with the cold steel beads method (75). Metabolite concentrations were analyzed by high-performance liquid chromatography (HPLC) on an Agilent 1100 HPLC (Agilent Technologies, Santa Clara, CA, USA) with an Aminex HPX-87H ion exchange column (Bio-Rad, Veenendaal, The Netherlands) operated at 60°C with 5 mM H_2_SO_4_ as the mobile phase at a flow rate of 0.6 ml min^−1^.

### Viability assays.

For cell viability determination based on membrane integrity (via propidium iodide [PI]), approximately 0.5 ml of culture broth was sampled into 15 ml of ice-cold 10 mM Na-HEPES buffer (pH 7.2) containing 2% (wt/vol) glucose and kept on ice. Cell concentrations were determined using a Z2 Coulter counter (Beckman Coulter, Fullerton, CA, USA) set to a detection interval of 1.5 to 5.8 μm. The buffered sample was then diluted in isotone II diluent (Beckman Coulter, Woerden, The Netherlands) to a suspension containing 10^7^ cells ml^−1^ and stained with PI (Sigma-Aldrich) as described previously (76). The stained samples were analyzed on an Accuri C6 flow cytometer (BD Biosciences, Franklin Lakes, NJ, USA), equipped with a 488-nm laser, and detected by the FL-3 channel (620-nm band pass filter) for PI staining. Per sample, 30,000 events (cells) were analyzed. The viability was determined using Flowing software, version 2.5.1 (Perrtu Terho, Turku Centre for Biotechnology, University of Turku, Finland), by subtracting the percentage of PI-stained cells from a starting value of 100%. For determination of cell viability based on CFU counts, cultures were sampled into Na-HEPES buffer as described above and analyzed on a BD FACSAria II SORP cell sorter (BD Biosciences, Franklin Lakes, NJ), equipped with a 70-μm nozzle and operated with filtered FACSFlow sheath fluid (BD Biosciences). Evaluation of cytometer performance, analysis of cell morphology, and cell sorting were essentially performed as described previously (77). Gating of cell populations for CFU count determination by plating was performed so that typically more than 90% of all detected events (cells) would be sorted. Viability was determined as the average percentage of sorted cells able to form a colony after 3 days of incubation at 30°C on quintuplicate YPD plates (96 cells sorted per plate).

### Calculation of growth rate dependency of maintenance energy requirements.

For chemostat cultures, specific growth rate (μ) and biomass-specific glucose uptake rate (*q_S_*) were calculated by solving biomass and substrate mass balances assuming steady-state conditions, and least squares linear regression was used to estimate maintenance energy substrate requirements (*m_S_*; intercept with *y*-axis) and theoretical maximum biomass yield (*Y_X_*_/_*_S_*^max^; reciprocal of slope) coefficients (26) from *q_S_*/μ relationships. Calculations for retentostat cultures were performed essentially as described by C. Rebnegger et al. (29): pairs of μ and *q_S_* values were calculated from biomass accumulation between adjacent sampling points, and *m_S_* values were estimated via least squares linear regression from moving windows of continuous pairs of calculated μ and *q_S_* values (including from chemostat cultivations), with the exception that moving windows of 5 *q_S_*-μ pairs were used for *m_S_* estimation.

### Whole-genome sequencing and stability of *NUBM* disruption.

Genomic DNA of CBS11895 and IMD010 was isolated using a Qiagen 100/G kit (Qiagen, Hilden, Germany) from a shake flask culture grown in YPD medium to stationary phase, according to the manufacturer’s instructions. DNA concentrations were quantified using a Qubit fluorometer, version 2.0 (Thermo Fisher Scientific). CBS11895 was sequenced by Novogene Bioinformatics Technology Co., Ltd. (Yuen Long, Hong Kong) on a HiSeq 2500 instrument (Illumina, San Diego, CA, USA) with 150-bp paired-end reads using a True-seq PCR-free library preparation (Illumina). IMD010 was sequenced on a MiSeq instrument (Illumina) using a TruSeq DNA PCR-free library preparation as described previously (78).

In order to verify the genetic stability of the *nubm^G445GC^* disruption in strain IMD010 during the prolonged glucose-limited cultivations, a minimum of four single-colony isolates from each individual chemostat and retentostat cultivation with IMD010 was tested for the presence of the mutation. To this end, the cultures were plated for single colonies on solid YPD medium at the last sampling point of each fermentation, their genomic DNA was isolated, and primers 12200 and 12201 were used to PCR amplify and Sanger sequence the site containing the *nubm*^G445GC^ disruption, as described above. The *nubm^G445GC^* genotype was still present in all investigated colonies, and no additional mutations were detected within any of the sequenced 688-bp amplicons.

### RNA extraction, RNA sequencing, and transcriptome data analysis.

Sampling for transcriptome analysis was performed by quenching culture broth directly into liquid nitrogen to immediately stop mRNA turnover (79), followed by storage at –80°C. In the case of batch cultures, sampling for transcriptome analysis was done in mid-exponential phase at a biomass dry weight concentration of approximately 0.9 g liter^−1^ with >75% of the initial glucose concentration remaining in the reactor. Processing of samples for long-term storage using AE buffer, acid-phenol-chloroform-isoamyl alcohol (125:24:1, pH 4.5; Thermo Fisher Scientific), and sodium dodecyl sulfate, as well as total RNA isolation was performed as described previously (77). The quality of the total extracted RNA was evaluated with an Agilent 2200 Tapestation (Agilent Technologies, Santa Clara, CA), and the RNA concentration was determined using a Qubit 2.0 fluorometer (Thermo Fisher Scientific) combined with a Qubit RNA BR (broad-range) assay kit (Thermo Fisher Scientific). Library preparation and RNA sequencing were performed by Novogene Bioinformatics Technology Co., Ltd. (Yuen Long, Hong Kong). Sequencing was done with an Illumina paired-end 150-bp sequencing read system (PE150) using a 250- to 300-bp insert strand-specific library which was prepared by Novogene. For the library preparation, mRNA enrichment was done using oligo(dT) beads. After random fragmentation of the mRNA, cDNA was synthetized from the mRNA using random hexamer primers. Afterwards, second-strand synthesis was done by addition of a custom second-strand synthesis buffer (Illumina), deoxynucleoside triphosphates (dNTPs), RNase H, and DNA polymerase I. Finally, after terminal repair, A ligation, and adaptor ligation, the double-stranded cDNA library was finalized by size selection and PCR enrichment.

The sequencing data for the samples obtained by Novogene had an average read depth of 21 million reads per sample. For each sample, reads were aligned to the genome of CBS11895 (DL-1) RefSeq assembly (NCBI accession number GCF_000187245.1) (67) with the two-pass STAR procedure (80). In the first pass, a splice junction database was assembled which was used to inform the second round of alignments. Introns were allowed to be between 15 and 4,000 bp, and soft clipping was disabled to prevent low-quality reads from being spuriously aligned. Ambiguously mapped reads were removed. Expression was quantified per transcript using HTSeq count in union intersection mode (81). To exclude from the analysis genes expressed at low levels, genes with an average fragments per kilobase per million (FPKM) value below 10 in all samples were removed. Counts were normalized by TMM normalization using the edgeR package (82), and subsequently differentially expressed genes were determined with an absolute log_2_ fold change of >2 and a false-discovery rate of < 0.001. Mean normalization of transcript data was performed per gene and separately for strains CBS11895 and IMD010 using the TMM-normalized FPKM values and was done either including (for comparative expression analysis) or excluding (for analysis of growth rate-correlated gene expression) data from the batch fermentations. For identification of growth rate-correlated gene clusters, analysis of the mean-normalized transcript values versus specific growth rate was performed using the maSigPro R package (83, 84). Genes with a trend significantly different from the mean were selected with a Benjamini-Hochberg corrected *P* value of <0.1, and subsequently the regression parameters for two clusters of genes were identified with a significance cutoff of 0.05 and an *R*^2^ of >0.8. For determination of enriched Gene Ontology (GO) terms in growth rate-correlated gene clusters and sets of differentially expressed genes, the online generic GO Term Finder tool (http://go.princeton.edu/cgi-bin/GOTermFinder) and *Saccharomyces* Genome Database annotation were used. A cutoff of 0.01 was used for the corrected *P* value (Bonferroni correction), and a background list was provided containing all O. parapolymorpha CBS11895 protein-coding genes for which S. cerevisiae S288C orthologs could be identified (3,094 out of 5,325; obtained using the Orthologous Matrix Database [85]). The number of GO terms was reduced using REVIGO with an allowed similarity setting of 0.5 (86) (see Data Set S1 in the supplemental material for all identified GO terms).

### Proteome processing and data analysis.

For proteome sampling, a culture sample equivalent to 2 to 4 mg of biomass dry weight was sampled into precooled microcentrifuge tubes, pelleted by centrifugation at 4°C at 4,700 × *g* for 5 min, and washed with 1.5 ml of ice-cold 1× phosphate-buffered saline (Sigma-Aldrich). After an additional centrifugation step under identical conditions and subsequent removal of the phosphate-buffered saline, cell pellets were stored at –80°C until further processing. In the case of batch cultures, sampling for proteome analysis was done in mid-exponential phase at a biomass dry weight concentration of approximately 0.9 g liter^−1^ with >75% of the initial glucose concentration remaining in the reactor. To process samples for analysis, cell mass was normalized to a dry weight of 1.6 mg and mechanically lysed using 0.5-mm zirconium beads and a PreCellys homogenizer. Proteins were isolated using Bligh and Dyer extraction (87), followed by reduction, alkylation, and digestion using trypsin. Samples were analyzed in technical triplicates by liquid chromatography tandem mass spectrometry (LC-MS/MS) using a Vanquish UHPLC coupled to a Q Exactive Plus Orbitrap MS (Thermo Fisher Scientific). Peptides were separated using reverse-phase chromatography using a gradient of water with 0.1% formic acid (solvent A) and acetonitrile with 0.1% formic acid (solvent B) from 2% B to 45% B in 50 min. Data-dependent acquisition (DDA) was performed with a resolution setting at 70,000 within the 400- to 1,600-*m/z* range and a maximum injection time of 75 ms, followed by high-energy collision-induced dissociation-activated (HCD) MS/MS on the top 15 most abundant precursors using a resolution setting of 17,500 and a 200- to 2,000-*m/z* range with a maximum injection time of 50 ms. The minimum intensity threshold for MS/MS was 1,000 counts, and peptide species with 1 and >8 charges were excluded. MS/MS spectra were analyzed with the SEQUEST HT search engine and Proteome Discoverer, version 2.3, against the proteins of the CBS11895 (DL-1) RefSeq assembly (NCBI accession no. GCF_000187245.1) (67). Label-free quantification was performed using the top three unique peptides measured for each protein. Retention time alignment was performed on the most abundant signals obtained from nonmodified peptides measured in all samples, and results were corrected for the total ion intensities measured for each sample. For subsequent analysis, only proteins were taken along that achieved a combined detection confidence with an FDR of <1% and additionally were individually detected with an FDR of <1% in at least 5 out of the total 48 LC-MS analyses (6 per condition). For proteins that passed these requirements, protein abundance was set to 0 for individual analyses that did not exhibit a detection confidence with an FDR of <1%, and the average abundance of all analyses per condition was used for further calculation. Proteins were considered not detected for a specific condition if they were not measured at least once with a detection confidence of an FDR of <1% for that condition. Mean normalization of the protein data was performed per gene and separately for strains CBS11895 and IMD010 using the total ion intensity-normalized protein abundances.

### Data availability.

Transcript abundances, lists of differentially expressed genes, sets of growth rate-correlated genes, identified S. cerevisiae orthologs of O. parapolymorpha protein-coding genes, complete lists of enriched GO terms, and total ion intensity-normalized protein abundances are available in Data Set S1 in the supplemental material.

Genome sequencing data of CBS11895 and IMD010 are available at NCBI (https://www.ncbi.nlm.nih.gov/) under BioProject accession number PRJNA588376. RNA-seq data are available at NCBI (https://www.ncbi.nlm.nih.gov/) under Gene Expression Omnibus (GEO) accession number GSE140480. Raw proteomics data are available on figshare (https://doi.org/10.6084/m9.figshare.11398773) (88).

## Supplementary Material

Supplemental file 1

Supplemental file 2

## References

[1] Cereghino JL, Cregg JM 2000 Heterologous protein expression in the methylotrophic yeast *Pichia pastoris*. FEMS Microbiol Rev 24:45–66. doi:10.1111/j.1574-6976.2000.tb00532.x. 10640598

[2] Löbs AK, Schwartz C, Wheeldon I 2017 Genome and metabolic engineering in non-conventional yeasts: current advances and applications. Synth Syst Biotechnol 2:198–207. doi:10.1016/j.synbio.2017.08.002. 29318200 PMC5655347

[3] van Dijk R, Faber KN, Kiel JA, Veenhuis M, van der Klei I 2000 The methylotrophic yeast *Hansenula polymorpha*: a versatile cell factory. Enzyme Microb Technol 26:793–800. doi:10.1016/s0141-0229(00)00173-3. 10862887

[4] Wagner JM, Alper HS 2016 Synthetic biology and molecular genetics in non-conventional yeasts: current tools and future advances. Fungal Genet Biol 89:126–136. doi:10.1016/j.fgb.2015.12.001. 26701310

[5] Vieira Gomes A, Souza Carmo T, Silva Carvalho L, Mendonça Bahia F, Parachin N 2018 Comparison of yeasts as hosts for recombinant protein production. Microorganisms 6:38. doi:10.3390/microorganisms6020038. PMC6027275 29710826

[6] Manfrao-Netto JHC, Gomes AMV, Parachin NS 2019 Advances in Using *Hansenula polymorpha* as chassis for recombinant protein production. Front Bioeng Biotechnol 7:94. doi:10.3389/fbioe.2019.00094. 31119131 PMC6504786

[7] Kunze G, Kang HA, Gellissen G 2009 *Hansenula polymorpha* (*Pichia angusta*): biology and applications, p 47–64. *In* Satyanarayana T, Kunze G (ed), Yeast biotechnology: diversity and applications. Springer, Dordrecht, Netherlands.

[8] Kurtzman CP 2011 *Ogataea* Y. Yamada, K. Maeda & M (1994), p 645–671. *In* Kurtzman CP, Fell JW, Boekhout T (ed), The yeasts: a taxonomic study, 5th ed Elsevier, London, United Kingdom.

[9] Juergens H, Niemeijer M, Jennings-Antipov LD, Mans R, Morel J, van Maris AJA, Pronk JT, Gardner TS 2018 Evaluation of a novel cloud-based software platform for structured experiment design and linked data analytics. Sci Data 5:180195. doi:10.1038/sdata.2018.195. 30280721 PMC6169258

[10] Hinkle PC 2005 P/O ratios of mitochondrial oxidative phosphorylation. Biochim Biophys Acta 1706:1–11. doi:10.1016/j.bbabio.2004.09.004. 15620362

[11] Ferguson SJ 2010 ATP synthase: from sequence to ring size to the P/O ratio. Proc Natl Acad Sci U S A 107:16755–16756. doi:10.1073/pnas.1012260107. 20858734 PMC2947903

[12] Kerscher SJ 2000 Diversity and origin of alternative NADH:ubiquinone oxidoreductases. Biochim Biophys Acta 1459:274–283. doi:10.1016/s0005-2728(00)00162-6. 11004440

[13] Kerscher S, Drose S, Zickermann V, Brandt U 2008 The three families of respiratory NADH dehydrogenases. Results Probl Cell Differ 45:185–222. doi:10.1007/400_2007_028. 17514372

[14] Joseph-Horne T, Hollomon DW, Wood PM 2001 Fungal respiration: a fusion of standard and alternative components. Biochim Biophys Acta 1504:179–195. doi:10.1016/s0005-2728(00)00251-6. 11245784

[15] Riley R, Haridas S, Wolfe KH, Lopes MR, Hittinger CT, Goker M, Salamov AA, Wisecaver JH, Long TM, Calvey CH, Aerts AL, Barry KW, Choi C, Clum A, Coughlan AY, Deshpande S, Douglass AP, Hanson SJ, Klenk HP, LaButti KM, Lapidus A, Lindquist EA, Lipzen AM, Meier-Kolthoff JP, Ohm RA, Otillar RP, Pangilinan JL, Peng Y, Rokas A, Rosa CA, Scheuner C, Sibirny AA, Slot JC, Stielow JB, Sun H, Kurtzman CP, Blackwell M, Grigoriev IV, Jeffries TW 2016 Comparative genomics of biotechnologically important yeasts. Proc Natl Acad Sci U S A 113:9882–9887. doi:10.1073/pnas.1603941113. 27535936 PMC5024638

[16] de Vries S, Marres CA 1987 The mitochondrial respiratory chain of yeast. Structure and biosynthesis and the role in cellular metabolism. Biochim Biophys Acta 895:205–239. doi:10.1016/s0304-4173(87)80003-4. 2849479

[17] Tarrio N, Diaz Prado S, Cerdan ME, Gonzalez Siso MI 2005 The nuclear genes encoding the internal (KlNDI1) and external (KlNDE1) alternative NAD(P)H:ubiquinone oxidoreductases of mitochondria from *Kluyveromyces lactis*. Biochim Biophys Acta 1707:199–210. doi:10.1016/j.bbabio.2004.12.008. 15863098

[18] Hunte C, Zickermann V, Brandt U 2010 Functional modules and structural basis of conformational coupling in mitochondrial complex I. Science 329:448–451. doi:10.1126/science.1191046. 20595580

[19] Kerscher SJ, Okun JG, Brandt U 1999 A single external enzyme confers alternative NADH:ubiquinone oxidoreductase activity in *Yarrowia lipolytica*. J Cell Sci 112:2347–2354. 10381390 10.1242/jcs.112.14.2347

[20] Duarte M, Peters M, Schulte U, Videira A 2003 The internal alternative NADH dehydrogenase of *Neurospora crassa* mitochondria. Biochem J 371:1005–1011. doi:10.1042/bj20021374. 12556227 PMC1223338

[21] Bridges HR, Grgic L, Harbour ME, Hirst J 2009 The respiratory complexes I from the mitochondria of two *Pichia* species. Biochem J 422:151–159. doi:10.1042/BJ20090492. 19459785

[22] González-Barroso MM, Ledesma A, Lepper S, Pérez-Magán E, Zaragoza P, Rial E 2006 Isolation and bioenergetic characterization of mitochondria from *Pichia pastoris*. Yeast 23:307–313. doi:10.1002/yea.1355. 16544272

[23] Katz R, Kilpatrick L, Chance B 1971 Acquisition and loss of rotenone sensitivity in *Torulopsis utilis*. Eur J Biochem 21:301–307. doi:10.1111/j.1432-1033.1971.tb01470.x. 5106112

[24] Schwitzguebel JP, Palmer JM 1982 Properties of mitochondria as a function of the growth stages of *Neurospora crassa*. J Bacteriol 149:612–619. doi:10.1128/JB.149.2.612-619.1982. 6460022 PMC216549

[25] Blondin B, Gonde P, Ratomahenina R, Arnaud A, Galzy P 1984 A study of cyanide-insensitive respiration in the genus *Dekkera* and *Brettanomyces*. Microbiol Immunol 28:637–644. doi:10.1111/j.1348-0421.1984.tb00717.x. 6482745

[26] Pirt SJ 1965 The maintenance energy of bacteria in growing cultures. Proc R Soc Lond B Biol Sci 163:224–231. doi:10.1098/rspb.1965.0069. 4378482

[27] Verduyn C, Stouthamer AH, Scheffers WA, van Dijken JP 1991 A theoretical evaluation of growth yields of yeasts. Antonie Van Leeuwenhoek 59:49–63. doi:10.1007/BF00582119. 2059011

[28] Correia K, Yu SM, Mahadevan R 2017 Reconstructing the evolution of metabolism in budding yeasts. bioRxiv https://www.biorxiv.org/content/10.1101/237974v1.

[29] Rebnegger C, Vos T, Graf AB, Valli M, Pronk JT, Daran-Lapujade P, Mattanovich D 2016 *Pichia pastoris* exhibits high viability and a low maintenance energy requirement at near-zero specific growth rates. Appl Environ Microbiol 82:4570–4583. doi:10.1128/AEM.00638-16. 27208115 PMC4984280

[30] Boender LG, de Hulster EA, van Maris AJ, Daran-Lapujade PA, Pronk JT 2009 Quantitative physiology of *Saccharomyces cerevisiae* at near-zero specific growth rates. Appl Environ Microbiol 75:5607–5614. doi:10.1128/AEM.00429-09. 19592533 PMC2737911

[31] Vos T, Hakkaart XD, de Hulster EA, van Maris AJ, Pronk JT, Daran-Lapujade P 2016 Maintenance-energy requirements and robustness of *Saccharomyces cerevisiae* at aerobic near-zero specific growth rates. Microb Cell Fact 15:111. doi:10.1186/s12934-016-0501-z. 27317316 PMC4912818

[32] Ercan O, Bisschops MM, Overkamp W, Jorgensen TR, Ram AF, Smid EJ, Pronk JT, Kuipers OP, Daran-Lapujade P, Kleerebezem M 2015 Physiological and transcriptional responses of different industrial microbes at near-zero specific growth rates. Appl Environ Microbiol 81:5662–5670. doi:10.1128/AEM.00944-15. 26048933 PMC4551249

[33] van Bodegom P 2007 Microbial maintenance: a critical review on its quantification. Microb Ecol 53:513–523. doi:10.1007/s00248-006-9049-5. 17333428 PMC1915598

[34] Fecke W, Sled VD, Ohnishi T, Weiss H 1994 Disruption of the gene encoding the NADH-binding subunit of NADH: ubiquinone oxidoreductase in *Neurospora crassa*. Formation of a partially assembled enzyme without FMN and the iron-sulphur cluster N-3. Eur J Biochem 220:551–558. doi:10.1111/j.1432-1033.1994.tb18655.x. 8125114

[35] Verduyn C 1991 Physiology of yeasts in relation to biomass yields. Antonie Van Leeuwenhoek 60:325–353. doi:10.1007/BF00430373. 1807201

[36] Chesbro W, Evans T, Eifert R 1979 Very slow growth of *Escherichia coli*. J Bacteriol 139:625–638. doi:10.1128/JB.139.2.625-638.1979. 378981 PMC216912

[37] Arbige M, Chesbro WR 1982 Very slow growth of *Bacillus polymyxa*: stringent response and maintenance energy. Arch Microbiol 132:338–344. doi:10.1007/BF00413386.

[38] Tappe W, Laverman A, Bohland M, Braster M, Rittershaus S, Groeneweg J, van Verseveld HW 1999 Maintenance energy demand and starvation recovery dynamics of *Nitrosomonas europaea* and *Nitrobacter winogradskyi* cultivated in a retentostat with complete biomass retention. Appl Environ Microbiol 65:2471–2477. doi:10.1128/AEM.65.6.2471-2477.1999. 10347029 PMC91364

[39] Feng Y, Li WF, Li J, Wang JW, Ge JP, Xu D, Liu YJ, Wu KQ, Zeng QY, Wu JW, Tian CL, Zhou B, Yang MJ 2012 Structural insight into the type-II mitochondrial NADH dehydrogenases. Nature 491:478–482. doi:10.1038/nature11541. 23086143

[40] Prömper C, Schneider R, Weiss H 1993 The role of the proton-pumping and alternative respiratory chain NADH:ubiquinone oxidoreductases in overflow catabolism of *Aspergillus niger*. Eur J Biochem 216:223–230. doi:10.1111/j.1432-1033.1993.tb18136.x. 8365409

[41] von Jagow G, Klingenberg M 1970 Pathways of hydrogen in mitochondria of *Saccharomyces carlsbergensis*. Eur J Biochem 12:583–592. doi:10.1111/j.1432-1033.1970.tb00890.x. 4314881

[42] Bakker BM, Overkamp KM, van Maris AJ, Kotter P, Luttik MA, van Dijken JP, Pronk JT 2001 Stoichiometry and compartmentation of NADH metabolism in *Saccharomyces cerevisiae*. FEMS Microbiol Rev 25:15–37. doi:10.1111/j.1574-6976.2001.tb00570.x. 11152939

[43] Antos-Krzeminska N, Jarmuszkiewicz W 2019 Alternative type II NAD(P)H dehydrogenases in the mitochondria of protists and fungi. Protist 170:21–37. doi:10.1016/j.protis.2018.11.001. 30553126

[44] Bakker BM, Bro C, Kotter P, Luttik MA, van Dijken JP, Pronk JT 2000 The mitochondrial alcohol dehydrogenase Adh3p is involved in a redox shuttle in *Saccharomyces cerevisiae*. J Bacteriol 182:4730–4737. doi:10.1128/jb.182.17.4730-4737.2000. 10940011 PMC111347

[45] Suwannarangsee S, Kim S, Kim OC, Oh DB, Seo JW, Kim CH, Rhee SK, Kang HA, Chulalaksananukul W, Kwon O 2012 Characterization of alcohol dehydrogenase 3 of the thermotolerant methylotrophic yeast *Hansenula polymorpha*. Appl Microbiol Biotechnol 96:697–709. doi:10.1007/s00253-011-3866-2. 22249723

[46] Suwannarangsee S, Oh DB, Seo JW, Kim CH, Rhee SK, Kang HA, Chulalaksananukul W, Kwon O 2010 Characterization of alcohol dehydrogenase 1 of the thermotolerant methylotrophic yeast *Hansenula polymorpha*. Appl Microbiol Biotechnol 88:497–507. doi:10.1007/s00253-010-2752-7. 20635082

[47] Wallrath J, Schmidt M, Weiss H 1991 Concomitant loss of respiratory chain NADH:ubiquinone reductase (complex I) and citric acid accumulation in *Aspergillus niger*. Appl Microbiol Biotechnol 36:76–81. doi:10.1007/BF00164702.

[48] Pfeiffer T, Schuster S, Bonhoeffer S 2001 Cooperation and competition in the evolution of ATP-producing pathways. Science 292:504–507. doi:10.1126/science.1058079. 11283355

[49] Molenaar D, van Berlo R, de Ridder D, Teusink B 2009 Shifts in growth strategies reflect tradeoffs in cellular economics. Mol Syst Biol 5:323. doi:10.1038/msb.2009.82. 19888218 PMC2795476

[50] Chen Y, Nielsen J 2019 Energy metabolism controls phenotypes by protein efficiency and allocation. Proc Natl Acad Sci U S A 116:17592–17597. doi:10.1073/pnas.1906569116. 31405984 PMC6717264

[51] Nilsson A, Bjornson E, Flockhart M, Larsen FJ, Nielsen J 2019 Complex I is bypassed during high intensity exercise. Nat Commun 10:5072. doi:10.1038/s41467-019-12934-8. 31699973 PMC6838197

[52] Marreiros BC, Sena FV, Sousa FM, Batista AP, Pereira MM 2016 Type II NADH:quinone oxidoreductase family: phylogenetic distribution, structural diversity and evolutionary divergences. Environ Microbiol 18:4697–4709. doi:10.1111/1462-2920.13352. 27105286

[53] Dominiak K, Koziel A, Jarmuszkiewicz W 2018 The interplay between mitochondrial reactive oxygen species formation and the coenzyme Q reduction level. Redox Biol 18:256–265. doi:10.1016/j.redox.2018.07.018. 30059902 PMC6078054

[54] Veiga A, Arrabaca JD, Loureiro-Dias MC 2003 Cyanide-resistant respiration, a very frequent metabolic pathway in yeasts. FEMS Yeast Res 3:239–245. doi:10.1016/S1567-1356(03)00036-9. 12689632

[55] Guerrero-Castillo S, Araiza-Olivera D, Cabrera-Orefice A, Espinasa-Jaramillo J, Gutiérrez-Aguilar M, Luévano-Martínez LA, Zepeda-Bastida A, Uribe-Carvajal S 2011 Physiological uncoupling of mitochondrial oxidative phosphorylation. Studies in different yeast species. J Bioenerg Biomembr 43:323–331. doi:10.1007/s10863-011-9356-5. 21556887

[56] Guerrero-Castillo S, Cabrera-Orefice A, Vázquez-Acevedo M, González-Halphen D, Uribe-Carvajal S 2012 During the stationary growth phase, *Yarrowia lipolytica* prevents the overproduction of reactive oxygen species by activating an uncoupled mitochondrial respiratory pathway. Biochim Biophys Acta 1817:353–362. doi:10.1016/j.bbabio.2011.11.007. 22138628

[57] Cabrera-Orefice A, Guerrero-Castillo S, Díaz-Ruíz R, Uribe-Carvajal S 2014 Oxidative phosphorylation in *Debaryomyces hansenii*: physiological uncoupling at different growth phases. Biochimie 102:124–136. doi:10.1016/j.biochi.2014.03.003. 24657599

[58] Guerrero-Castillo S, Baertling F, Kownatzki D, Wessels HJ, Arnold S, Brandt U, Nijtmans L 2017 The assembly pathway of mitochondrial respiratory chain complex I. Cell Metab 25:128–139. doi:10.1016/j.cmet.2016.09.002. 27720676

[59] Stroud DA, Surgenor EE, Formosa LE, Reljic B, Frazier AE, Dibley MG, Osellame LD, Stait T, Beilharz TH, Thorburn DR, Salim A, Ryan MT 2016 Accessory subunits are integral for assembly and function of human mitochondrial complex I. Nature 538:123–126. doi:10.1038/nature19754. 27626371

[60] Schägger H, Pfeiffer K 2000 Supercomplexes in the respiratory chains of yeast and mammalian mitochondria. EMBO J 19:1777–1783. doi:10.1093/emboj/19.8.1777. 10775262 PMC302020

[61] Nubel E, Wittig I, Kerscher S, Brandt U, Schagger H 2009 Two-dimensional native electrophoretic analysis of respiratory supercomplexes from *Yarrowia lipolytica*. Proteomics 9:2408–2418. doi:10.1002/pmic.200800632. 19343715

[62] Marques I, Dencher NA, Videira A, Krause F 2007 Supramolecular organization of the respiratory chain in *Neurospora crassa* mitochondria. Eukaryot Cell 6:2391–2405. doi:10.1128/EC.00149-07. 17873079 PMC2168242

[63] Eubel H, Heinemeyer J, Sunderhaus S, Braun HP 2004 Respiratory chain supercomplexes in plant mitochondria. Plant Physiol Biochem 42:937–942. doi:10.1016/j.plaphy.2004.09.010. 15707832

[64] Hirst J 2018 Open questions: respiratory chain supercomplexes-why are they there and what do they do? BMC Biol 16:111. doi:10.1186/s12915-018-0577-5. 30382836 PMC6211484

[65] Matus-Ortega MG, Cárdenas-Monroy CA, Flores-Herrera O, Mendoza-Hernández G, Miranda M, González-Pedrajo B, Vázquez-Meza H, Pardo JP 2015 New complexes containing the internal alternative NADH dehydrogenase (Ndi1) in mitochondria of *Saccharomyces cerevisiae*. Yeast 32:629–641. doi:10.1002/yea.3086. 26173916

[66] Lõoke M, Kristjuhan K, Kristjuhan A 2011 Extraction of genomic DNA from yeasts for PCR-based applications. Biotechniques 50:325–328. doi:10.2144/000113672. 21548894 PMC3182553

[67] Ravin NV, Eldarov MA, Kadnikov VV, Beletsky AV, Schneider J, Mardanova ES, Smekalova EM, Zvereva MI, Dontsova OA, Mardanov AV, Skryabin KG 2013 Genome sequence and analysis of methylotrophic yeast *Hansenula polymorpha* DL1. BMC Genomics 14:837. doi:10.1186/1471-2164-14-837. 24279325 PMC3866509

[68] Souciet J, Aigle M, Artiguenave F, Blandin G, Bolotin-Fukuhara M, Bon E, Brottier P, Casaregola S, de Montigny J, Dujon B, Durrens P, Gaillardin C, Lépingle A, Llorente B, Malpertuy A, Neuvéglise C, Ozier-Kalogéropoulos O, Potier S, Saurin W, Tekaia F, Toffano-Nioche C, Wésolowski-Louvel M, Wincker P, Weissenbach J 2000 Genomic exploration of the hemiascomycetous yeasts: 1. A set of yeast species for molecular evolution studies. FEBS Lett 487:3–12. doi:10.1016/s0014-5793(00)02272-9. 11152876

[69] Bridges HR, Fearnley IM, Hirst J 2010 The subunit composition of mitochondrial NADH:ubiquinone oxidoreductase (complex I) from *Pichia pastoris*. Mol Cell Proteomics 9:2318–2326. doi:10.1074/mcp.M110.001255. 20610779 PMC2953923

[70] Eldarov MA, Mardanov AV, Beletsky AV, Ravin NV, Skryabin KG 2011 Complete sequence and analysis of the mitochondrial genome of the methylotrophic yeast *Hansenula polymorpha* DL-1. FEMS Yeast Res 11:464–472. doi:10.1111/j.1567-1364.2011.00736.x. 21545683

[71] Juergens H, Varela JA, Gorter de Vries AR, Perli T, Gast VJM, Gyurchev NY, Rajkumar AS, Mans R, Pronk JT, Morrissey JP, Daran JG 2018 Genome editing in *Kluyveromyces* and *Ogataea* yeasts using a broad-host-range Cas9/gRNA co-expression plasmid. FEMS Yeast Res 18:foy012. doi:10.1093/femsyr/foy012. PMC6018904 29438517

[72] Verduyn C, Postma E, Scheffers WA, Van Dijken JP 1992 Effect of benzoic acid on metabolic fluxes in yeasts: a continuous-culture study on the regulation of respiration and alcoholic fermentation. Yeast 8:501–517. doi:10.1002/yea.320080703. 1523884

[73] Postma E, Verduyn C, Scheffers WA, Van Dijken JP 1989 Enzymic analysis of the Crabtree effect in glucose-limited chemostat cultures of *Saccharomyces cerevisiae*. Appl Environ Microbiol 55:468–477. doi:10.1128/AEM.55.2.468-477.1989. 2566299 PMC184133

[74] Verduyn C, Postma E, Scheffers WA, van Dijken JP 1990 Physiology of *Saccharomyces cerevisiae* in anaerobic glucose-limited chemostat cultures. J Gen Microbiol 136:395–403. doi:10.1099/00221287-136-3-395. 1975265

[75] Mashego MR, van Gulik WM, Vinke JL, Heijnen JJ 2003 Critical evaluation of sampling techniques for residual glucose determination in carbon-limited chemostat culture of *Saccharomyces cerevisiae*. Biotechnol Bioeng 83:395–399. doi:10.1002/bit.10683. 12800134

[76] Boender LG, Almering MJ, Dijk M, van Maris AJ, de Winde JH, Pronk JT, Daran-Lapujade P 2011 Extreme calorie restriction and energy source starvation in *Saccharomyces cerevisiae* represent distinct physiological states. Biochim Biophys Acta 1813:2133–2144. doi:10.1016/j.bbamcr.2011.07.008. 21803078

[77] Brickwedde A, Brouwers N, van den Broek M, Gallego Murillo JS, Fraiture JL, Pronk JT, Daran JG 2018 Structural, physiological and regulatory analysis of maltose transporter genes in *Saccharomyces eubayanus* CBS 12357(T). Front Microbiol 9:1786. doi:10.3389/fmicb.2018.01786. 30147677 PMC6097016

[78] Swiat MA, Dashko S, den Ridder M, Wijsman M, van der Oost J, Daran JM, Daran-Lapujade P 2017 FnCpf1: a novel and efficient genome editing tool for *Saccharomyces cerevisiae*. Nucleic Acids Res 45:12585–12598. doi:10.1093/nar/gkx1007. 29106617 PMC5716609

[79] Piper MD, Daran-Lapujade P, Bro C, Regenberg B, Knudsen S, Nielsen J, Pronk JT 2002 Reproducibility of oligonucleotide microarray transcriptome analyses. An interlaboratory comparison using chemostat cultures of *Saccharomyces cerevisiae*. J Biol Chem 277:37001–37008. doi:10.1074/jbc.M204490200. 12121991

[80] Dobin A, Davis CA, Schlesinger F, Drenkow J, Zaleski C, Jha S, Batut P, Chaisson M, Gingeras TR 2013 STAR: ultrafast universal RNA-seq aligner. Bioinformatics 29:15–21. doi:10.1093/bioinformatics/bts635. 23104886 PMC3530905

[81] Anders S, Pyl PT, Huber W 2015 HTSeq—a Python framework to work with high-throughput sequencing data. Bioinformatics 31:166–169. doi:10.1093/bioinformatics/btu638. 25260700 PMC4287950

[82] Robinson MD, McCarthy DJ, Smyth GK 2010 edgeR: a Bioconductor package for differential expression analysis of digital gene expression data. Bioinformatics 26:139–140. doi:10.1093/bioinformatics/btp616. 19910308 PMC2796818

[83] Conesa A, Nueda MJ, Ferrer A, Talon M 2006 maSigPro: a method to identify significantly differential expression profiles in time-course microarray experiments. Bioinformatics 22:1096–1102. doi:10.1093/bioinformatics/btl056. 16481333

[84] Nueda MJ, Tarazona S, Conesa A 2014 Next maSigPro: updating maSigPro bioconductor package for RNA-seq time series. Bioinformatics 30:2598–2602. doi:10.1093/bioinformatics/btu333. 24894503 PMC4155246

[85] Altenhoff AM, Glover NM, Train CM, Kaleb K, Warwick Vesztrocy A, Dylus D, de Farias TM, Zile K, Stevenson C, Long J, Redestig H, Gonnet GH, Dessimoz C 2018 The OMA orthology database in 2018: retrieving evolutionary relationships among all domains of life through richer web and programmatic interfaces. Nucleic Acids Res 46:D477–D485. doi:10.1093/nar/gkx1019. 29106550 PMC5753216

[86] Supek F, Bosnjak M, Skunca N, Smuc T 2011 REVIGO summarizes and visualizes long lists of gene ontology terms. PLoS One 6:e21800. doi:10.1371/journal.pone.0021800. 21789182 PMC3138752

[87] Sapcariu SC, Kanashova T, Weindl D, Ghelfi J, Dittmar G, Hiller K 2014 Simultaneous extraction of proteins and metabolites from cells in culture. MethodsX 1:74–80. doi:10.1016/j.mex.2014.07.002. 26150938 PMC4472845

[88] Juergens H, Hakkaart XDV, Bras JE, Vente A, Wu L, Benjamin KR, Pronk JT, Daran-Lapujade P, Mans R 2020 Proteomics data of glucose-grown *Ogataea parapolymorpha*. figshare 10.6084/m9.figshare.11398773. PMC7376551 32471916

