# Supplementary material for: Contribution of Complex I NADH Dehydrogenase to Respiratory Energy Coupling in Glucose-Grown Cultures of Ogataea parapolymorpha
Source: Appl Environ Microbiol. 2020 Jul 20;86(15):e00678-20. doi: 10.1128/AEM.00678-20 (PMC7376551; doi:10.1128/AEM.00678-20)
Supplement: Supplemental file 1 [file AEM.00678-20-s0001.pdf]

Supplementary material

Table S1: Physiology of wild type strain *O. parapolymorpha* CBS11895 and Complex I-disrupted mutant IMD010 in aerobic glucose-limited chemostat cultures at a dilution rate of 0.025 h^{-1}. Reported values are mean ± SEM calculated from two independent steady state cultures. Reported *p*-values (Student's *t*-test) refer to the difference between mean values observed for CBS11895 and IMD010. Carbon recovery calculations are based on a biomass carbon content of 48% (w/w). BDL: below detection limit (10 µM). Reported cell viability based on propidium iodide staining. Symbols: $Y_{X/S}$ and $Y_{X/O2}$ = yield of biomass dry weight on glucose and oxygen, respectively; RQ = respiratory quotient; $q_{Glucose}$, q_{CO2} and q_{O2} = biomass-specific uptake/production rate of glucose, CO_2 and O_2, respectively; C_X = biomass dry weight concentration. The presented data was determined from chemostat cultures preceding retentostat cultivations, with exception of the biomass protein content, which was determined from separate, duplicate chemostat cultures.

**Strain**	**CBS11895**	**IMD010**	***p*-value**
Actual dilution rate (h^{-1})	0.025 ± 0.000	0.025 ± 0.000	-
Reservoir glucose (g L^{-1})	10.03 ± 0.01	9.75 ± 0.05	-
Residual glucose (mM)	BDL	BDL	-
$Y_{X/S}$ (g biomass [g glucose]$^{-1}$)	0.42 ± 0.00	0.33 ± 0.00	<.001
$Y_{X/O2}$ (g biomass [g O_2]$^{-1}$)	0.78 ± 0.03	0.52 ± 0.01	.030
RQ	0.97 ± 0.02	1.00 ± 0.03	.38
$q_{Glucose}$ (mmol [g biomass]$^{-1}$ h^{-1})	-0.34 ± 0.00	-0.42 ± 0.01	.044
q_{CO2} (mmol [g biomass]$^{-1}$ h^{-1})	0.98 ± 0.01	1.53 ± 0.02	.011
q_{O2} (mmol [g biomass]$^{-1}$ h^{-1})	-1.02 ± 0.03	-1.52 ± 0.06	.033
C_X (g biomass L^{-1})	4.20 ± 0.01	3.24 ± 0.00	.007
Protein content (g protein [g biomass]$^{-1}$)	0.36 ± 0.01	0.39 ± 0.02	.26

Cell viability (%)	99.4 ± 0.2	99.8 ± 0.1	.20
Carbon recovery (%)	99.0 ± 0.7	100.0 ± 0.7	.41

Table S2: Enriched GO terms (p value cutoff 0.01; Bonferroni correction) of categories 'biological process' (BP), 'molecular function' (MF) and 'cellular component' (CC) found in set of significantly upregulated genes in *O. parapolymorpha* strain IMD010 (devoid of functional Complex I) compared to CBS11895 (wild type) in aerobic glucose-grown batch cultures. GO enrichment analysis was based on 275 (out of 409) genes for which an *S. cerevisiae* ortholog could be identified, and a background (genome) of 3095 genes was used for testing. Redundant GO terms were removed using REVIGO. See data appendix for full list of GO terms.

GO term	Type	Description	P value (corrected)	Genes in set	Genes in genome
GO:0022613	BP	ribonucleoprotein complex biogenesis	5.99E-18	89	381
GO:0010467	BP	gene expression	6.24E-17	162	1033
GO:0044085	BP	cellular component biogenesis	1.42E-15	135	796
GO:0006364	BP	rRNA processing	3.40E-12	57	219
GO:0002181	BP	cytoplasmic translation	2.18E-09	37	121
GO:0034641	BP	cellular nitrogen compound metabolic process	1.02E-08	177	1395
GO:0006396	BP	RNA processing	6.60E-08	73	397
GO:0043603	BP	cellular amide metabolic process	3.77E-06	70	406
GO:0034660	BP	ncRNA metabolic process	4.82E-06	67	383
GO:0071840	BP	cellular component organization or biogenesis	1.50E-05	165	1363
GO:0043170	BP	macromolecule metabolic process	2.57E-04	181	1592
GO:0043933	BP	macromolecular complex subunit organization	5.45E-04	72	474
GO:0044271	BP	cellular nitrogen compound biosynthetic process	2.49E-03	113	891
GO:0033108	BP	mitochondrial respiratory chain complex assembly	4.62E-03	12	31
GO:0003735	MF	structural constituent of ribosome	2.23E-23	58	146
GO:0005198	MF	structural molecule activity	8.80E-18	62	205
GO:0003723	MF	RNA binding	1.09E-04	67	427
GO:0019843	MF	rRNA binding	4.98E-04	17	55
GO:0003676	MF	nucleic acid binding	5.71E-03	88	677
GO:1990904	CC	ribonucleoprotein complex	1.03E-28	116	462
GO:0044391	CC	ribosomal subunit	1.81E-21	58	156
GO:0043228	CC	non-membrane-bounded organelle	1.03E-14	138	850
GO:0032991	CC	macromolecular complex	1.83E-13	186	1381
GO:0031974	CC	membrane-enclosed lumen	2.60E-12	125	776
GO:0005730	CC	Nucleolus	3.98E-12	56	218
GO:0098798	CC	mitochondrial protein complex	1.05E-08	41	156
GO:0044445	CC	cytosolic part	1.89E-06	36	148
GO:0044428	CC	nuclear part	5.91E-06	99	683
GO:0044446	CC	intracellular organelle part	1.38E-05	205	1848
GO:0005688	CC	U6 snRNP	1.86E-05	6	6
GO:0005732	CC	small nucleolar ribonucleoprotein complex	1.27E-04	9	19
GO:1990726	CC	Lsm1-7-Pat1 complex	3.44E-03	5	6

Table S3: Enriched GO terms (p value cutoff 0.01; Bonferroni correction) of categories 'biological process' (BP), 'molecular function' (MF) and 'cellular component' (CC) found in sets of genes that exhibited positive or negative correlation of expression with specific growth rate in *O. parapolymorpha* CBS11895 (wild type) and/or IMD010 (devoid of functional Complex I), based on samples taken from glucose-limited chemostat (0.1 and 0.025 h^{-1}) and late-stage retentostat (0.001 h^{-1}) cultures. Test sets: a (positive correlation only in CBS11895; 459 genes), b (positive correlation in both CBS11895 and IMD010; 414 genes), c (positive correlation only in IMD010; 169 genes), d(negative correlation only in CBS11895; 137 genes). All gene sets were tested against a background (genome) of 3095 genes. Redundant GO terms were removed using REVIGO. See data appendix for full list of GO terms.

Set	GO term	Description	Type	P value (corrected)	Genes in set	Genes in genome
a	GO:0051276	chromosome organization	BP	1.14E-05	84	317
a	GO:0007059	chromosome segregation	BP	1.33E-04	35	96
a	GO:0098813	nuclear chromosome segregation	BP	1.12E-03	29	78
a	GO:0000280	nuclear division	BP	3.49E-03	35	108
a	GO:0071824	protein-DNA complex subunit organization	BP	5.62E-03	35	110
a	GO:0097159	organic cyclic compound binding	MF	1.94E-03	216	1164
a	GO:0003677	DNA binding	MF	4.32E-03	65	266
a	GO:1901363	heterocyclic compound binding	MF	5.10E-03	213	1158
a	GO:0005488	binding	MF	5.79E-03	316	1857
a	GO:0015630	microtubule cytoskeleton	CC	4.00E-06	27	59
a	GO:0043228	non-membrane-bounded organelle	CC	5.38E-04	169	850
a	GO:0000794	condensed nuclear chromosome	CC	8.34E-04	19	42
a	GO:0005856	cytoskeleton	CC	1.32E-03	39	128
a	GO:0044428	nuclear part	CC	3.66E-03	138	683
a	GO:0005634	nucleus	CC	7.87E-03	229	1269
b	GO:0009058	biosynthetic process	BP	1.29E-07	231	1274
b	GO:1901576	organic substance biosynthetic process	BP	1.32E-07	230	1267
b	GO:0044281	small molecule metabolic process	BP	8.22E-07	112	558
b	GO:0006520	cellular amino acid metabolic process	BP	9.12E-05	54	198
b	GO:0017144	drug metabolic process	BP	3.21E-03	48	186
b	GO:0003824	catalytic activity	MF	6.24E-05	263	1602
b	GO:0071162	CMG complex	CC	4.19E-03	8	11
c	GO:0140101	catalytic activity, acting on a tRNA	MF	2.14E-03	15	76
d	GO:0016021	integral component of membrane	CC	1.87E-10	64	632
d	GO:0005783	endoplasmic reticulum	CC	1.55E-08	47	413
d	GO:0012505	endomembrane system	CC	4.65E-07	60	675
d	GO:0016020	membrane	CC	3.03E-06	81	1121
d	GO:0044444	cytoplasmic part	CC	3.54E-05	115	1999
d	GO:0031984	organelle subcompartment	CC	3.56E-05	37	356
δ	GO:0098827	endoplasmic reticulum subcompartment	CC	2.14E-04	29	259
δ	GO:0005737	cytoplasm	CC	2.16E-03	129	2529

Table S4: Protein abundances of nuclearly encoded Complex I subunits and alternative NADH dehydrogenases (NDH2) in *O. parapolymorpha* strains CBS11895 (wild type) and IMD010 (disrupted Complex I Nubm subunit). Samples were taken from aerobic glucose-grown batch (0.37 h^{-1}), chemostat (0.1 and 0.025 h^{-1}) and retentostat (0.001 h^{-1}) cultures. Data is presented as mean ± standard deviation of two independent biological replicate analyses (each based on three technical replicates). The number between parentheses denotes how many times (out of six technical replicates) a specific protein was detected with a detection confidence of FDR <1%. N.D.: 'not detected' (see Methods section). The Complex I subunits were assigned based on sequence homology with subunits from *Pichia pastoris* (69). For *P. pastoris* Complex I subunits NB5M, NB8M and NUTM, corresponding ORFs can be detected in the genome of *O. parapolymorpha*, but were not annotated as protein-coding in the *O. parapolymorpha* reference assembly (67) and excluded from analysis. Complex I subunit NUUM (HPODL_05121) was excluded from proteome analysis because abundance data did not pass quality requirements for analysis (see Methods section).

			CBS11895				IMD010			
**Subunit**	**Locus tag**	**Type**	**0.37 h^{-1}**	**0.1h^{-1}**	**0.025 h^{-1}**	**0.001 h^{-1}**	**0.37 h^{-1}**	**0.1h^{-1}**	**0.025 h^{-1}**	**0.001 h^{-1}**
NUAM	HPODL_03689	Essential	179824 ± 38% (6)	481529 ± 0% (6)	442908 ± 25% (6)	322146 ± 30% (6)	96960 ± 12% (5)	266627 ± 9% (6)	30351 ± 7% (4)	N.D.
NUBM	HPODL_04625	Essential	71482 ± 56% (6)	280305 ± 2% (6)	316559 ± 5% (6)	293642 ± 31% (6)	N.D.	N.D.	N.D.	N.D.
NUCM	HPODL_01297	Essential	24688 (1)	375650 ± 2% (6)	344104 ± 10% (6)	144700 ± 67% (4)	N.D.	353512 ± 5% (6)	211590 ± 1% (6)	N.D.
NUGM	HPODL_03393	Essential	50286 (2)	399470 ± 0% (6)	370648 ± 4% (6)	203294 ± 39% (5)	15278 (1)	337246 ± 2% (6)	93404 ± 67% (3)	N.D.
NUHM	HPODL_01287	Essential	32053 ± 41% (2)	394675 ± 3% (6)	384382 ± 13% (6)	242639 ± 14% (6)	N.D.	N.D.	N.D.	N.D.
NUIM	HPODL_02101	Essential	N.D.	77726 ± 54% (3)	148803 ± 16% (6)	14726 (1)	N.D.	N.D.	20701 ± 11% (2)	N.D.
NUKM	HPODL_02758	Essential	8047 (1)	206143 ± 4% (6)	202897 ± 15% (6)	34087 ± 22% (2)	N.D.	106113 ± 1% (6)	23979 ± 28% (2)	N.D.
NUEM	HPODL_03913	Accessory	25354 ± 47% (2)	359541 ± 15% (6)	382660 ± 16% (6)	277320 ± 3% (6)	47377 ± 35% (2)	254346 ± 1% (6)	191010 ± 7% (6)	N.D.
NESM	HPODL_04326	Accessory	N.D.	329988 ± 4% (6)	285892 ± 6% (6)	29033 (1)	N.D.	354216 ± 5% (6)	226425 ± 4% (6)	N.D.
NUJM	HPODL_01263	Accessory	N.D.	251225 ± 11% (6)	257097 ± 9% (6)	126511 ± 25% (5)	N.D.	238167 ± 11% (6)	160546 ± 29% (6)	N.D.
NUXM	HPODL_00687	Accessory	N.D.	79080 (2)	86825 ± 16% (2)	15807 (1)	N.D.	113479 ± 65% (3)	33902 (1)	N.D.
NUPM	HPODL_04116	Accessory	42052 ± 28% (2)	235964 ± 28% (5)	218736 ± 10% (5)	29678 (1)	47353 ± 24% (2)	336688 ± 3% (6)	170028 ± 22% (5)	N.D.
NUZM	HPODL_02157	Accessory	23774 (1)	405364 ± 0% (6)	432082 ± 8% (6)	313045 ± 14% (6)	N.D.	282122 ± 4% (6)	138582 ± 84% (4)	N.D.
NUSM	HPODL_00962	Accessory	5151 (1)	357940 ± 25% (5)	416528 ± 9% (6)	166118 ± 85% (4)	N.D.	462020 ± 3% (6)	135084 ± 61% (3)	5393 (1)
NIMM	HPODL_04436	Accessory	N.D.	184355 ± 31% (5)	138416 ± 81% (4)	21623 (1)	N.D.	206603 ± 29% (5)	74854 ± 20% (3)	N.D.
NB6M	HPODL_04286	Accessory	N.D.	95476 ± 42% (3)	77662 ± 34% (3)	22259 (1)	N.D.	94476 ± 40% (3)	24656 (1)	N.D.
N7BM	HPODL_02876	Accessory	N.D.	207768 ± 25% (5)	236554 ± 0% (6)	21041 (1)	N.D.	N.D.	N.D.	N.D.
NUYM	HPODL_02906	Accessory	N.D.	284591 ± 5% (6)	273960 ± 11% (6)	175143 ± 49% (5)	N.D.	N.D.	N.D.	N.D.
NIAM	HPODL_04805	Accessory	30291 ± 29% (2)	279664 ± 0% (6)	150171 ± 45% (5)	19912 (1)	28892 ± 20% (2)	292267 ± 0% (6)	88641 ± 48% (3)	N.D.
NUMM	HPODL_01160	Accessory	N.D.	146881 ± 10% (6)	136240 ± 1% (6)	37833 ± 44% (3)	3085 (1)	16775 (2)	4790 (1)	N.D.
NUFM	HPODL_00771	Accessory	N.D.	242389 ± 14% (6)	302979 ± 2% (6)	181680 ± 18% (5)	N.D.	204788 ± 1% (6)	81068 ± 26% (3)	N.D.
NB4M	HPODL_02851	Accessory	N.D.	145115 ± 6% (6)	158945 ± 12% (6)	15871 (1)	N.D.	16112 (1)	25179 ± 7% (2)	N.D.
NI2M	HPODL_05260	Accessory	62507 (2)	346886 ± 1% (6)	387578 ± 17% (6)	237959 ± 15% (5)	42787 ± 44% (2)	417024 ± 17% (6)	245943 ± 4% (6)	N.D.
NIPM	HPODL_02838	Accessory	N.D.	182097 ± 72% (4)	125963 ± 52% (3)	24355 (1)	N.D.	116294 ± 67% (3)	84215 ± 52% (3)	N.D.
ACPM2	HPODL_02246	Accessory	N.D.	540289 ± 5% (6)	499646 ± 11% (6)	161965 ± 82% (3)	N.D.	517691 ± 16% (6)	381346 ± 2% (6)	259593 ± 38% (5)
NIDM	HPODL_04828	Accessory	N.D.	363610 ± 4% (6)	327785 ± 4% (6)	6049 (1)	9165 ± 6% (2)	397009 ± 18% (6)	196454 ± 33% (5)	N.D.
NI8M	HPODL_04214	Accessory	N.D.	343986 ± 2% (6)	298730 ± 10% (6)	28227 (1)	32510 ± 0% (2)	200145 ± 19% (6)	27715 ± 18% (2)	N.D.
ACPM1	HPODL_01821	Accessory	618989 ± 9% (6)	883525 ± 6% (6)	761495 ± 1% (6)	581073 ± 23% (6)	719983 ± 4% (6)	815408 ± 18% (6)	734720 ± 2% (6)	754365 ± 6% (6)
NI9M	HPODL_05148	Accessory	23012 (1)	287906 ± 23% (5)	292372 ± 8% (6)	87698 ± 76% (3)	41804 ± 26% (2)	335671 ± 25% (6)	168099 ± 15% (4)	12915 (1)
NDH2-1	HPODL_02792	NDH2	274434 ± 37% (6)	N.D.	N.D.	N.D.	203027 ± 36% (5)	55633 ± 60% (3)	50900 ± 83% (3)	N.D.
NDH2-2	HPODL_00256	NDH2	540086 ± 0% (6)	337718 ± 11% (6)	490208 ± 21% (6)	467389 ± 4% (6)	517421 ± 5% (6)	295604 ± 5% (6)	394630 ± 0% (6)	450323 ± 7% (6)
NDH2-3	HPODL_02018	NDH2	118201 ± 2% (6)	N.D.	20315 ± 87% (3)	44509 ± 34% (4)	112193 ± 3% (6)	N.D.	7744 (1)	50213 ± 31% (5)

Figure S1

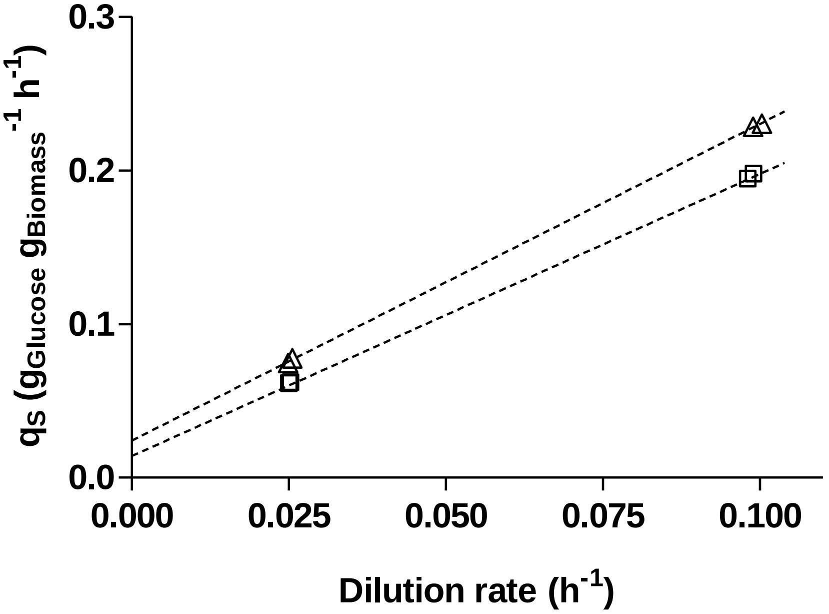

Figure S1: Biomass-specific glucose uptake rate (q_S) of wild type *O. parapolymorpha* CBS11895 (squares) and Complex I-deficient strain IMD010 (triangles) in aerobic, glucose-limited chemostat cultures. Each data point represents an independent biological replicate. Least squares linear regression (dashed lines) was used to estimate maintenance energy requirements (m_S; intercept with y axis) and theoretical maximum biomass yield ($Y_{X/S}^{max}$; reciprocal of slope) coefficients.

Figure S2

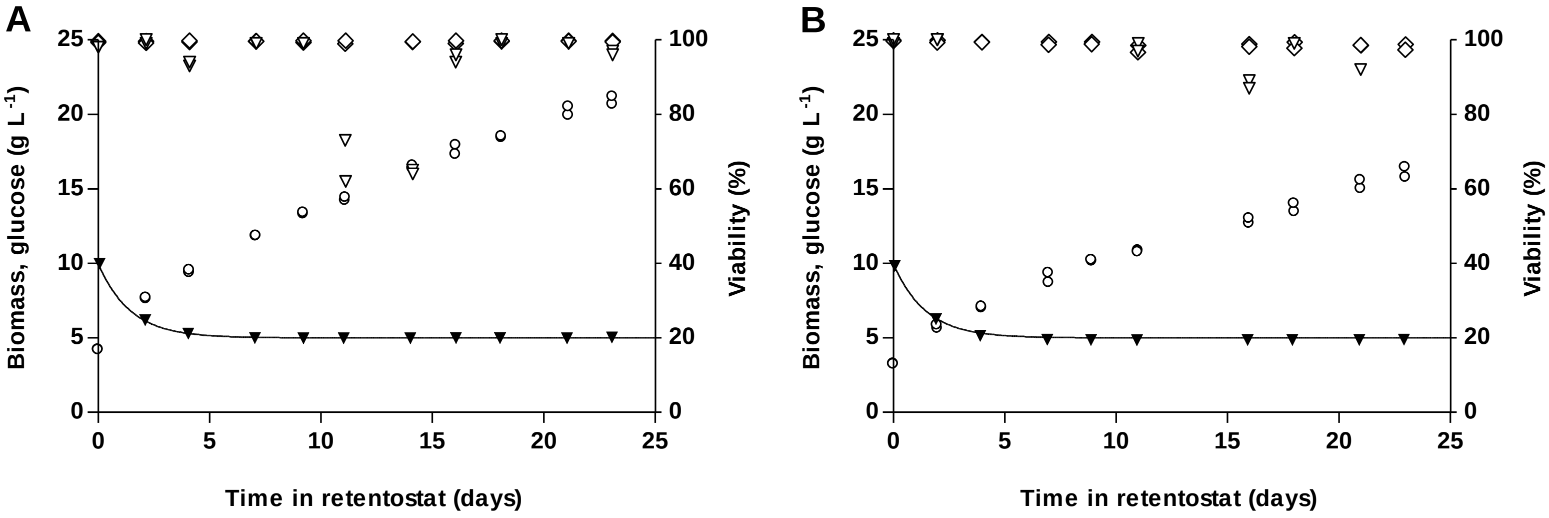

Figure S2: Biomass accumulation profile, viability and feed glucose concentration of aerobic glucose-limited *O. parapolymorpha* retentostat cultures of wild-type strain CBS11895 (A) and the congenic Complex I-deficient mutant IMD010 (B). Depicted are the measured biomass dry weight concentration (open circles), culture viability based on propidium iodide staining (open diamonds) and CFU determination (open triangles), and predicted (solid line) and measured (closed triangles) feed glucose concentration in the mixing vessel of two independent cultures from each strain.

Figure S3

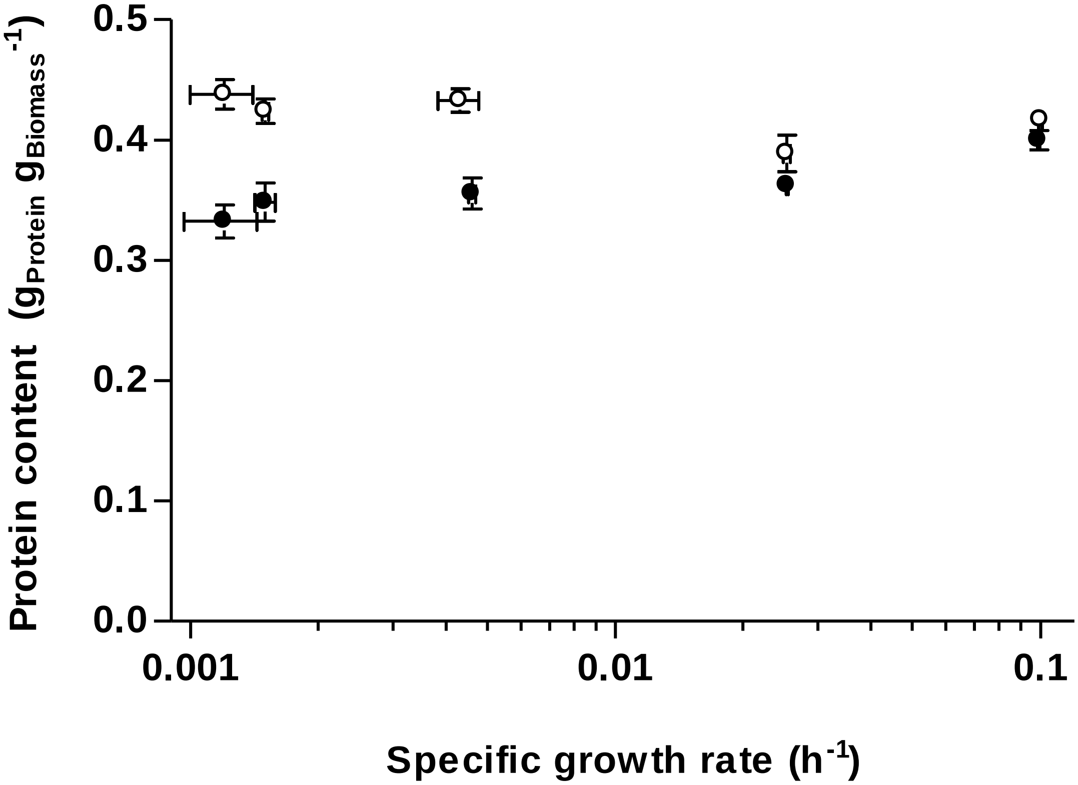

Figure S3: Biomass protein content of *O. parapolymorpha* strains CBS11895 (wild type; closed circles) and IMD010 (Complex I-deficient; open circles) at different specific growth rates. Samples were taken from steady state aerobic glucose-limited chemostat cultures (specific growth rates of 0.1 h^{-1} and 0.025 h^{-1}) and from aerobic glucose-limited retentostat cultures after 4, 11 and 21 days of cultivation (specific growth rates below 0.025 h^{-1}). For each data point samples were taken from two independent cultures, and data is presented as mean ± standard deviation. Horizontal error bars represent standard deviation of specific growth rate determined for retentostat cultures. For retentostat culture samples, the mean protein content of CBS11895 was found to be

significantly lower than that of IMD010 at each equivalent sampling point (Student's *t*-test, $p < .05$).

Figure S4

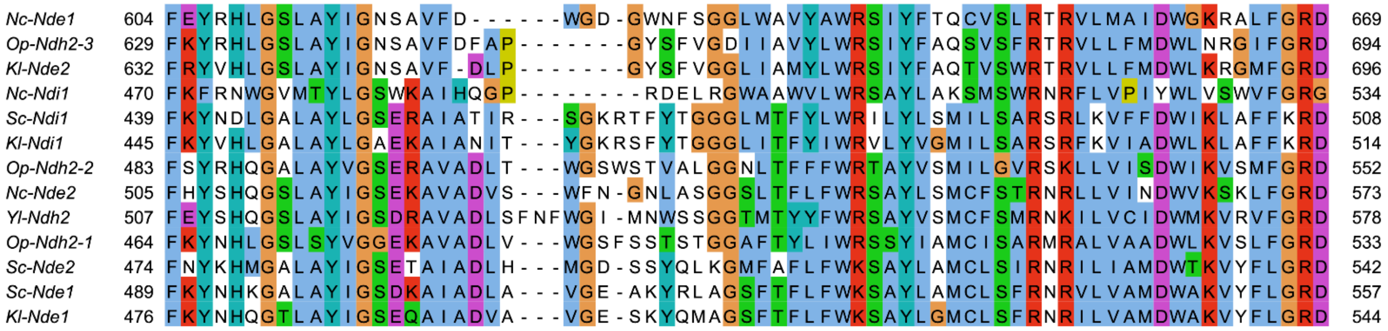

Figure S4: Section of MUSCLE sequence alignment showing the characteristic conserved C-terminal domain (39) of the three putative *O. parapolymorpha* alternative NAD(P)H dehydrogenases and known fungal/yeast alternative NAD(P)H dehydrogenases. Visualized by Jalview using Clustalx residue colors. *Kl*, *Kluyveromyces lactis*, *Nc*, *Neurospora crassa*, *Op*, *Ogataea parapolymorpha*, *Sc*, *Saccharomyces cerevisiae*, *Yl*, *Yarrowia lipolytica*.

Figure S5

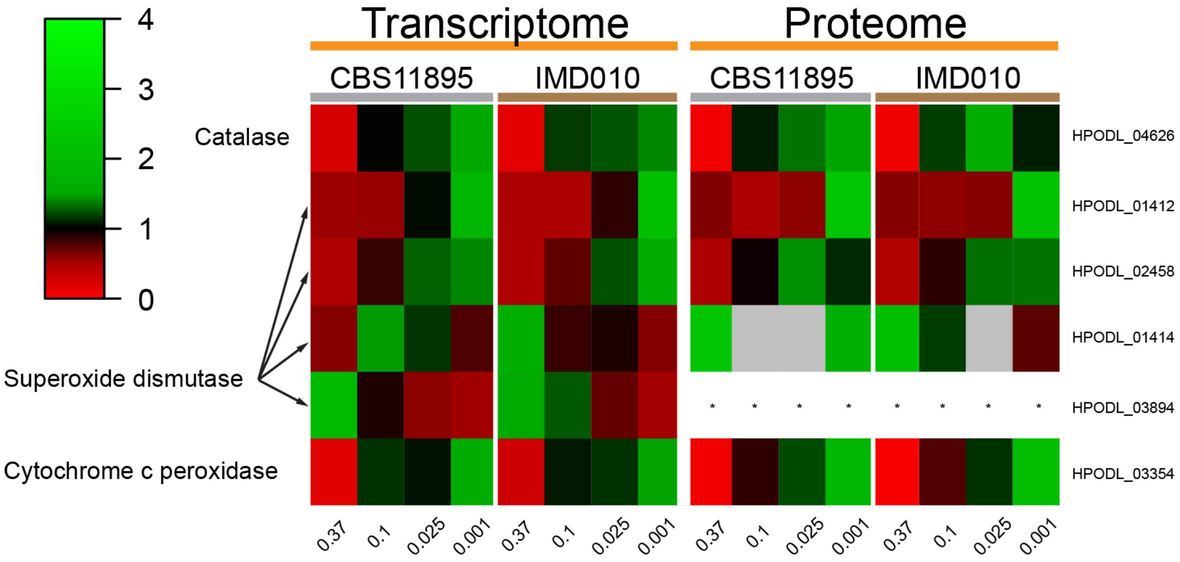

Figure S5: Mean-normalized transcript and protein abundances of genes related to detoxification of reactive oxygen species in *O. parapolymorpha* strains CBS11895 (wild type) and IMD010 (disrupted Complex I Nubm subunit). Samples were taken from duplicate independent aerobic glucose-grown batch (0.37 h^{-1}), chemostat (0.1 and 0.025 h^{-1}) and late-stage retentostat (0.001 h^{-1}) cultures. Transcript and protein abundances were mean-normalized separately for each gene and strain. Grey color: protein not detected based on criteria described in Methods section. Annotation of *O. parapolymorpha* catalase and superoxide dismutases was taken from (67), and HPODL_03354 was identified as ortholog of *S. cerevisiae* cytochrome c peroxidase (*CCP1*) via the orthologous matrix database (see methods section). *HPODL_03894 was excluded from proteome analysis because abundance data did not pass quality requirements for analysis (see Methods section).

References

39. Feng Y, Li WF, Li J, Wang JW, Ge JP, Xu D, Liu YJ, Wu KQ, Zeng QY, Wu JW, Tian CL, Zhou B, Yang MJ. 2012. Structural insight into the type-II mitochondrial NADH dehydrogenases. Nature 491:478-82.

67. Ravin NV, Eldarov MA, Kadnikov VV, Beletsky AV, Schneider J, Mardanova ES, Smekalova EM, Zvereva MI, Dontsova OA, Mardanov AV, Skryabin KG. 2013. Genome sequence and analysis of methylotrophic yeast *Hansenula polymorpha* DL1. BMC Genomics 14:837.

69. Bridges HR, Fearnley IM, Hirst J. 2010. The subunit composition of mitochondrial NADH:ubiquinone oxidoreductase (complex I) from *Pichia pastoris*. Mol Cell Proteomics 9:2318-26.
